# Genetic Determinants of Pelvic Organ Prolapse among African American and Hispanic Women in the Women’s Health Initiative

**DOI:** 10.1371/journal.pone.0141647

**Published:** 2015-11-06

**Authors:** Ayush Giri, Jennifer M. Wu, Renee M. Ward, Katherine E. Hartmann, Amy J. Park, Kari E. North, Mariaelisa Graff, Robert B. Wallace, Gihan Bareh, Lihong Qi, Mary J. O'Sullivan, Alexander P. Reiner, Todd L. Edwards, Digna R. Velez Edwards

**Affiliations:** 1 Institute for Medicine and Public Health, Vanderbilt Epidemiology Center, Vanderbilt University, Nashville, Tennessee, United States of America; 2 Department of Obstetrics and Gynecology, Center for Women’s Health Research, University of North Carolina, Chapel Hill, North Carolina, United States of America; 3 Department of Obstetrics and Gynecology, Vanderbilt University Medical Center, Nashville, Tennessee, United States of America; 4 Department of Medicine, Vanderbilt University Medical Center, Nashville, Tennessee, United States of America; 5 Department of Obstetrics and Gynecology and Urology, Georgetown University Medical Center, Washington, DC, United States of America; 6 Department of Epidemiology, University of North Carolina, Chapel Hill, North Carolina, United States of America; 7 Department of Epidemiology, University of Iowa, Iowa City, Iowa, United States of America; 8 Department of Obstetrics and Gynecology, Loma Linda University, Loma Linda, California, United States of America; 9 Department of Public Health Sciences, University of California, Davis, California, United States of America; 10 Department of Obstetrics and Gynecology, University of Miami, Miami, Florida, United States of America; 11 Department of Epidemiology, University of Washington, Seattle, Washington, United States of America; 12 Vanderbilt Genetics Institute, Vanderbilt University, Nashville, Tennessee, United States of America; Case Western Reserve University, UNITED STATES

## Abstract

Current evidence suggests a multifactorial etiology to pelvic organ prolapse (POP), including genetic predisposition. We conducted a genome-wide association study of POP in African American (AA) and Hispanic (HP) women from the Women’s Health Initiative Hormone Therapy study. Cases were defined as any POP (grades 1–3) or moderate/severe POP (grades 2–3), while controls had grade 0 POP. We performed race-specific multiple logistic regression analyses between SNPs imputed to 1000 genomes in relation to POP (grade 0 vs 1–3; grade 0 vs 2–3) adjusting for age at diagnosis, body mass index, parity, and genetic ancestry. There were 1274 controls and 1427 cases of any POP and 317 cases of moderate/severe POP. Although none of the analyses reached genome-wide significance (p<5x10^-8^), we noted variants in several loci that met p<10^−6^. In race-specific analysis of grade 0 vs 2–3, intronic SNPs in the *CPE* gene (rs28573326, OR:2.14; 95% CI 1.62–2.83; p = 1.0x10^-7^) were associated with POP in AAs, and SNPs in the gene *AL132709*.*5* (rs1950626, OR:2.96; 95% CI 1.96–4.48, p = 2.6x10^-7^) were associated with POP in HPs. Inverse variance fixed-effect meta-analysis of the race-specific results showed suggestive signals for SNPs in the *DPP6* gene (rs11243354, OR:1.36; p = 4.2x10^-7^) in the grade 0 vs 1–3 analyses and for SNPs around *PGBD5* (rs740494, OR:2.17; p = 8.6x10^-7^) and *SHC3* (rs2209875, OR:0.60; p = 9.3x10^-7^) in the grade 0 vs 2–3 analyses. While we did not identify genome-wide significant findings, we document several SNPs reaching suggestive statistical significance. Further interrogation of POP in larger minority samples is warranted.

## Introduction

Pelvic organ prolapse (POP) is a highly prevalent condition associated with significant morbidity that affects up to 40% of postmenopausal women [1], with 20% of women opting for surgical management by age 80 [2]. Although several risk factors for POP have been identified, including age [3], race [4;5], parity [1;6] and obesity [1;7;8], the underlying pathophysiology of POP remains unknown.

Epidemiologic studies have reported family history as a significant risk factor [9–11] and familial forms of POP have been reported [12;13]. In addition, several candidate gene studies [14–18] and one genome wide association study (GWAS) have identified promising single nucleotide polymorphisms (SNPs) associated with POP [19]. A recently published genome-wide linkage analysis provided evidence for two additional loci in relation to symptomatic POP [20]. However, a majority of the existing studies have focused on women of European ancestry and there are few validated loci known for POP [21].

Given the limitations in the current literature, the Women’s Health Initiative SNP Health Associated Resources (SHARe) dataset provides a unique opportunity to explore the genetic susceptibility to POP in minority women, as this dataset includes extensive phenotypic and genotypic information on African American (AA) and Hispanic (HP) postmenopausal women. Investigating the genetic epidemiology of POP in these populations is particularly important, as epidemiologic data suggest that the risk for POP varies by race. Prior studies have reported that AA women have the lowest risk [1;5], while white and Hispanic women have the highest risks [1;5]. Thus, the objective of our study was to identify loci associated with POP in AA and HP women using the Women’s Health Initiative-SHARe dataset.

## Materials and Methods

### Study Population

Data used in this study were obtained from AA and HP women who were enrolled in the Women’s Health Initiative hormone therapy (HT) trial (registered in ClinicalTrials.gov; registration number: NCT00000611) and for whom genotyping data was available through Women’s Health Initiative-SHARe. The Women’s Health Initiative is a large prospective study which recruited 161,861 post-menopausal women between 50–79 years of age from 40 clinical centers throughout the US from 1993–1998. Eligible participants were enrolled to the observational study or one or more of the three clinical trials: HT, dietary modification and/or calcium and Vitamin D supplementation trial [22]. Briefly, post-menopausal women who were unlikely to move and had predicted survival for three or more years, who were not using hormone therapy or were willing to stop, and who were currently not participating in any other clinical trial were eligible to participate.

Information regarding demographics, clinical, behavioral characteristics, medical history, and lifestyle/behavioral factors, among other risk factors, was obtained by standardized self-administered questionnaires at baseline. Information regarding POP was ascertained through standardized pelvic exams performed on women participating in the Women’s Health Initiative-HT trial. Baseline pelvic exams were performed using standardized procedures by a gynecologist, experienced nurse, or physician assistant. Pelvic exams included an evaluation of the presence of uterine prolapse, cystocele, and rectocele using Women’s Health Initiative Prolapse Classification System (grades: 0, no prolapse; 1, prolapse in vagina; 2, prolapse to introitus; and 3, prolapse outside vagina). Examination for POP was performed in a supine lithotomy position while participant was asked to perform the Valsalva maneuver. Annual pelvic exams were also performed during follow-up physical exams with participants having between one to 10 visits. Further details regarding Women’s Health Initiative protocols and ascertainment for Women’s Health Initiative have been previously described [23;24].

For this study, cases were defined as women with a cystocele, rectocoele, or uterine prolapse of any severity (grades 1–3) at baseline or at one of the 10 follow-up visits. Since multiple assessments of POP were available for women in this study, the first occurrence of a cystocele, rectocele or uterine prolapse of any severity (grades 1–3) was coded as any POP, and the first occurrence of a cystocele, rectocele or uterine prolapse of grade II or higher was coded as moderate-severe POP. For women with multiple assessments of POP through time, the highest grade of POP across visits was used as their POP status for the purposes of our analyses. Controls were women who did not have any of the three forms of POP: rectocele, cystocele and uterine prolapse (grade 0) at baseline or at follow-up visits when POP was assessed. Our primary analyses compared controls to women with any POP (grade 0 vs. grade 1–3) or moderate/severe POP (grade 0 vs. grade 2–3). Variables examined in this study included age at diagnosis and variables collected at baseline: age at baseline interview, parity, body mass index (BMI), waist circumference, cigarette smoking history, prior hysterectomy, and menopausal hormone use status. De-identified data were assessed through authorized and secure methods after Institutional Review Board approval at Vanderbilt University and approval from the Women’s Health Initiative, and the database of Genotypes and Phenotypes (dbGaP).

### Genotyping

The Affymetrix Human SNP Array 6.0 (Affymetrix®, Inc Santa Clara, CA) was used for genome wide SNP genotyping. Genomic DNA was quantitated via an ND-8000 spectrophotometer and DNA quality was evaluated via gel electrophoresis. The genomic DNA samples were processed according to standard Affymetrix procedures for processing of the assay. The data were processed for genotype calling using the Affymetrix® Genotypic Console software using the Birdseed calling algorithm version 2.0 (Affymetrix®, Inc., Santa Clara, CA) [25].

### Quality Control (QC)

Data on 909,622 SNPs and 12,007 individuals were available prior to implementation of quality control. 177 individuals were removed after excluding individuals with low genotyping quality (<98%). 188 individuals with first degree or higher relatedness were identified using identity-by-descent sharing from a random selection of 100,000 autosomal SNPs, and removed from analysis. Finally, we removed 140 subjects with inconsistent reported versus genetically determined sex. This resulted in 8,180 AA and 3,322 HP women remaining after sample QC.

**Fig 1:**
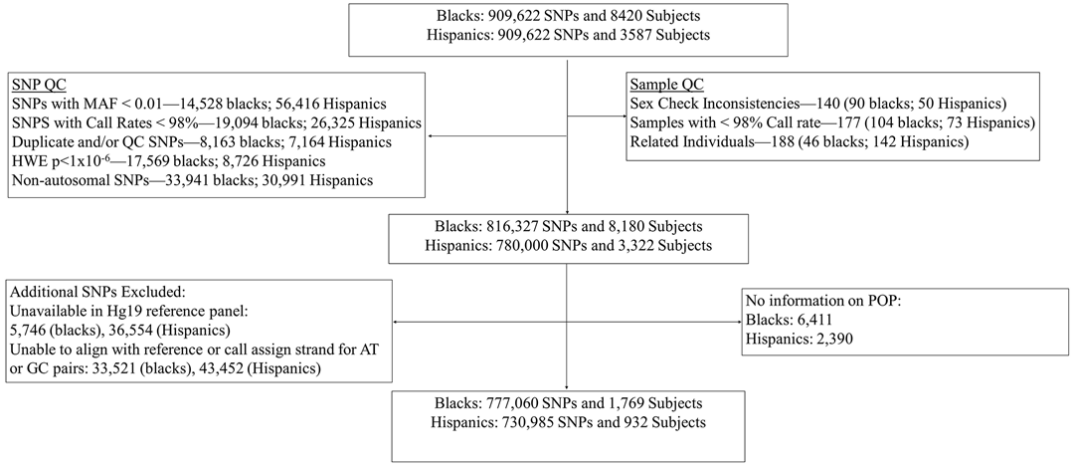
The flow chart presents an overview of sample and SNP inclusion/exclusion during quality control.

All SNPs were tested for deviation from Hardy-Weinberg equilibrium (HWE) using PLINK software, stratified by race [26]. We excluded SNPs with HWE *P*≤10^−6^, low genotyping quality (<98%), a minor allele frequency (MAF) < 0.01, non-autosomal SNPs, and SNPs that did not map to a chromosomal position. Furthermore, we plotted the allele frequencies for the AA and HP datasets against the allele frequencies corresponding to the African reference population and American Indian reference population from the 1000 genomes build 37, respectively. We created these plots to identify and exclude SNPs which had discordant allele frequencies (absolute value difference > 0.1) compared with their respective reference populations, and to exclude palindromic SNPs with MAF > 0.4 for which the reference strand could not be determined easily. This resulted in a total of 777,060 SNPs in AAs and 730,985 SNPs in HPs available for imputation. Quality control procedures are detailed in Fig 1.

Imputation was conducted using IMPUTE2, using samples and genotypes that passed QC and all 1000 genomes reference panels (build 37, 2013) to increase imputation accuracy [27;28]. Imputation was limited to 1769 AAs and 932 HPs, as data on POP was limited to these individuals. Prior to imputation, genotype data were phased using SHAPEIT software [29].

### Analytic Sample

Of the 8180 AA and 3322 HP women with genetic data, 1,769 AA and 932 HP women had ≥1 pelvic exams and thus contributed information on POP. Of the 1,769 AA and 932 HP women, 514 AA and 437 HP women had POP (considering any severity of rectocele, cystocele and uterine prolapse) at baseline. During follow-up, 292 AA women and 184 HP women additionally developed POP. Our final analytical sample included 793 grades 1–3 cases, 154 grades 2–3 cases, and 948 controls in AA women, and 606 grades 1–3 cases, 163 grades 2–3 cases, and 305 controls in HP women who had no missing data on important covariates such as age at diagnosis, body mass index, hysterectomy status and parity.

### Population Stratification

In order to assess population stratification among AAs and HP samples multi-dimensional scaling (MDS) was employed using PLINK software to estimate continuous axes of ancestry [26]. The top four MDS components were extracted for AAs and HP groups individually and used as covariates in SNP association analyses to adjust for potential confounding due to ancestry. A plot of the top two MDS components from the sample data (AAs and HP), and the International HapMap Project Phase 3 populations (ASW: African ancestry in Southwest USA; MXL: Mexican ancestry from Los Angeles, CA; LWK: Luhya in Webuye, Kenya; MKK: Maasai in Kinyawa, Kenya; YRI: Yoruban in Ibadan, Nigeria) was produced to visualize ancestral genetic differences (S1 Fig).

### Power

We performed power calculations *a priori* using the Quanto (Version 1.2.4) software. Assuming a meta-analysis sample size of approximately 1400 cases, a genome-wide p-value threshold of 5 x 10^−8^, an approximate 1:1 case to control ratio, a baseline prevalence of 40% and a log-additive model for SNP effect, we estimated 80% power to detect minimum odds ratios of 1.48, 1.45, 1.43, and 1.42 for minor allele frequencies of 0.25, 0.30, 0.35 and 0.40, respectively.

### Statistical Analyses

Participant and demographic characteristics were analyzed with two-sample t-tests assuming unequal variance to compare between racial groups when variables were continuous, and Chi-squared tests when variables were categorical using Stata, version 11 (StataCorp, College Station, TX, USA).

**Fig 2:**
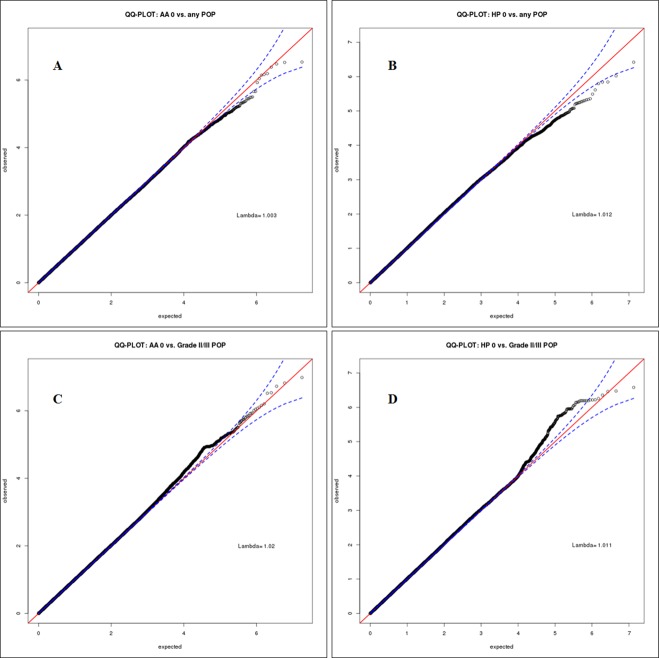
Quantile-quantile (Q-Q) plot of association analysis results. Expected–log10 p values are on the x-axis and the observed–log10 p values are on the y-axis. A) African Americans grade 0 vs. 1–3; B) Hispanics grade 0 vs. 1–3; C) African Americans grade 0 vs. 2&3; D) Hispanics grade 0 vs. 2&3.

The association between each genetic marker and POP status (binary) was assessed using logistic regression stratified by race adjusting for established POP risk factors identified in the literature including: age at POP ascertainment (continuous), BMI (continuous), parity (continuous) and MDS-derived axes of ancestry (continuous) using SNPTEST software [30]. Interpretation of analyses from logistic regression models were limited to genotyped and imputed SNPs with a post-imputation information score of ≥0.4, Hardy-Weinberg equilibrium P value >1x10^-8^, and minor allele frequency >0.05. Quantile-quantile plots for SNP association analyses for AA and HP subjects considering grade -0 vs. 1–3 and grade 0 vs. 2–3 POP are shown in Fig 2. Manhattan plots for race/ethnicity specific analyses considering grade -0 vs. 1–3 and grade 0 vs. 2–3 POP are shown in S2 Fig through S5 Fig. We then conducted inverse variance weighted fixed-effect meta-analysis between AA and HP results using the PLINK software. We resorted to using a fixed-effect meta-analysis technique instead of more sophisticated trans-ethnic meta-analysis techniques such as new random-effect models [31] or MANTRA [32] since only two independent datasets were available.

We performed several sensitivity analyses. We compared the results of six genome-wide significant SNPs reported by a previous GWAS study on POP with our results [19]. In the WHI-HT a large proportion of women had hysterectomy at baseline, with no indication for the reason for hysterectomy and the possibility that some of these women may have had POP previously could not be ruled out. Consequently, in the WHI-HT hysterectomy status was inversely associated with POP. However, women without a uterus may still develop other forms of POP such as rectocele and cystocele. In order to minimize the possibility that the associations observed with SNPs were not directed by any potential bias introduced by this misclassification, top hits (p < 10^−5^) from primary models were additionally adjusted for hysterectomy status. We also performed logistic regression analyses by strata of hysterectomy status for the top-SNPs from primary models. All p-values presented are two-sided.

## Results

In this case-control study, the mean ages at diagnosis for AA and HP women were 62 and 60 years respectively (Table 1). Characteristics of AA and HP participants from this sub-study are similar to those in the parent Women’s Health Initiative-HT study. Compared with HP women, AA women were on average more likely to be older, have higher BMI (29.8 vs. 31.6), more likely to be current smokers, and more likely to have had prior hysterectomy at baseline (40.7% vs. 57.7%). Advancing age, increasing BMI and parity were all associated with increased odds of POP across all grades (S1 Table).Prior hysterectomy was inversely associated with POP (OR: 0.24; 95% CI: 0.20, 0.29). HP women were approximately twice as likely as AA women to have POP (S1 Table). The racial disparity in POP existed irrespective of hysterectomy status.

**Table 1:** Demographic Characteristics of African American (AA) and Hispanic (HP) POP cases and controls from the Women’s Health Initiative.

**Continuous Variables**	**AA (N = 1769)**			**HP (N = 932)**	
	**Cases (806)**	**Controls (963)**		**Cases (621)**	**Controls (311)**
	**Mean (SD)**	**Mean (SD)**		**Mean (SD)**	**Mean (SD)**
**Age at baseline (years)**	61.84 (6.96)	61.13 (7.01)		59.62 (6.35)	59.62 (6.32)
**Age at diagnosis (years)** *	63.12 (7.09)	61.14 (7.01)		60.37 (6.45)	60.42 (6.43)
**BMI (kg/m** ^**2**^ **)** *	31.62 (6.18)	31.51 (6.44)		29.89 (5.27)	29.68 (5.89)
**Waist Circumference (cm)**	93.56 (13.03)	92.70 (13.35)		89.33 (11.87)	88.46 (12.55)
**Categorical Variables**	**N (%)**	**N (%)**		**N (%)**	**N (%)**
**BMI**					
<25 kg/m2	100 (12.41)	143 (14.85)		107 (17.23)	65 (20.90)
25-<30 kg/m2	258 (32.01)	293 (30.43)		239 (38.49)	121 (38.91)
> = 30 kg/m2	446 (55.33)	521 (54.10)		274 (44.12)	122 (39.23)
Missing	2 (0.25)	6 (0.62)		1 (0.16)	3 (0.93)
**Parity** *					
Nulliparous	81 (10.05)	163 (16.93)		39 (6.28)	31 (9.97)
1	91 (11.29)	149 (15.47)		44 (7.09)	32 (10.29)
2	181 (22.46)	211 (21.91)		115 (18.52)	63 (20.26)
3	143 (17.74)	171 (17.76)		109 (17.55)	66 (21.22)
4	107 (13.28)	99 (16.72)		119 (19.16)	44 (14.15)
≥5	192 (23.82)	161 (16.72)		184 (29.63)	72 (23.15)
Missing	11 (1.36)	9 (0.93)		11 (1.77)	3 (0.96)
**Hysterectomy**					
No	463 (57.44)	286 (29.70)		452 (72.79)	101 (32.48)
Yes	343 (42.56)	677 (70.30)		169 (27.21)	210 (67.52)
**Smoking status**					
Never	393 (48.76)	431 (44.76)		396 (63.77)	184 (59.16)
Past	298 (36.97)	361 (37.49)		162 (26.09)	98 (31.51)
Current	103 (12.78)	149 (15.47)		54 (8.70)	29 (9.32)
Missing	12 (1.49)	22 (2.28)		9 (1.45)	0 (0.00)
**Hormone therapy use**					
Never	459 (56.95)	511 (53.06)		366 (58.94)	163 (52.41)
Past	281 (34.86)	362 (37.59)		184 (29.63)	113 (36.33)
Current	51 (6.33)	86 (8.93)		54 (8.70)	34 (10.93)
Missing	15 (1.86)	4 (0.42)		17 (2.74)	1 (0.32)

*Primary logistic regression models were adjusted for the following demographic variables: age at ascertainment, BMI (continuous) and parity continuous)

**Fig 3:**
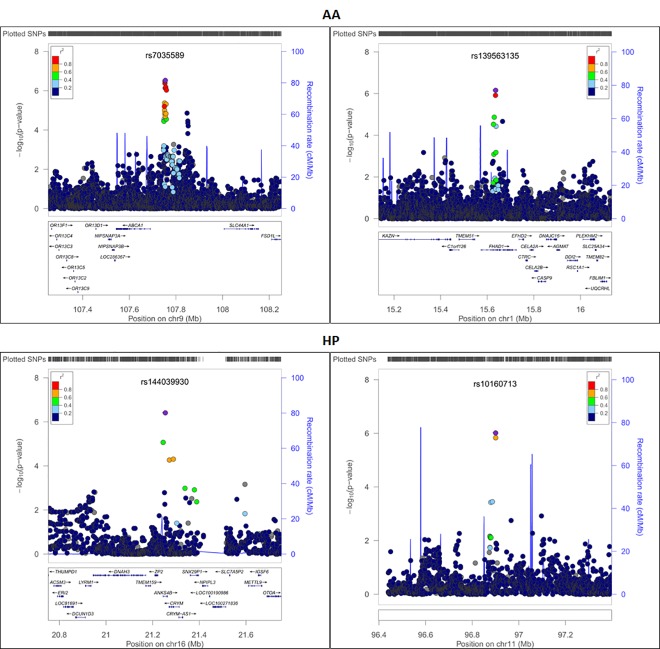
Regional association plots showing–log10(p-values) against base-pair position for African American (AA) (top) and Hispanic (HP) (bottom) women considering grade 0 vs. 1–3 analyses.

We first evaluated the relationships between SNPs and any severity of POP in AA and HP populations separately using multiple logistic regression models adjusted for age at ascertainment, BMI, parity and four MDS components of genetic ancestry (Table 2). While no SNPs reached genome-wide significance, we noted those that reached a P value threshold of <10^−6^. In analyses specific to AA women considering any POP, SNP rs7035589 (OR: 1.44; 95% CI: 1.25–1.65; P = 2.92x10^-7^), located near the gene ATP-binding cassette, subfamily-A (ABC1), member 1 (*ABCA1*) had the smallest P value. Similarly, in analyses specific to HP women considering any POP, SNP rs144039930 (OR: 0.15; 95% CI: 0.07–0.31; P = 3.80x10^-7^) located in the intronic region of gene ankyrin repeat and sterile alpha motif domain containing 4B (*ANKS4B*) had the smallest P value. Regional association plots corresponding to these loci are presented in Fig 3.

**Table 2:** Top genetic loci associated with any pelvic organ prolapse (grade 0 vs. 1–3) in African American (AA) and Hispanic (HP) women from the Women’s Health Initiative.

	SNP	CHR	BP	On/Nearby Genes	EA/RA	EAF	info	OR	95% CI	P
**AA**	rs7035589	9	107752358	*LOC101928579* *,*ABCA1*	T/C	0.39	1.00	1.44	1.25–1.65	2.92x10^-7^
	rs139563135	1	15633817	*FHAD1* *	A/G	0.15	0.81	1.64	1.35–2.00	6.82x10^-7^
**HP**	rs144039930	16	21252260	*ANKS4B* *,*ZP2*,*CRYM*	T/C	0.05	0.68	0.15	0.07–0.31	3.80x10^-7^
	rs10160713	11	96898237	*LOC100131233*,*MAML2*	T/A	0.06	0.77	0.22	0.12–0.40	9.51x10^-7^

CHR = Chromosome; SNP = Single Nucleotide Polymorphism; EA = Effect Allele; RA = Reference Allele

*SNP is on gene

EAF = Effect Allele Frequency for Controls; OR = Odds Ratio; CI = Confidence Interval; P = P-value from logistic regression; Logistic regression models were adjusted for age at ascertainment, BMI (continuous), parity continuous) and 4 genetic ancestry components (continuous)

**Fig 4:**
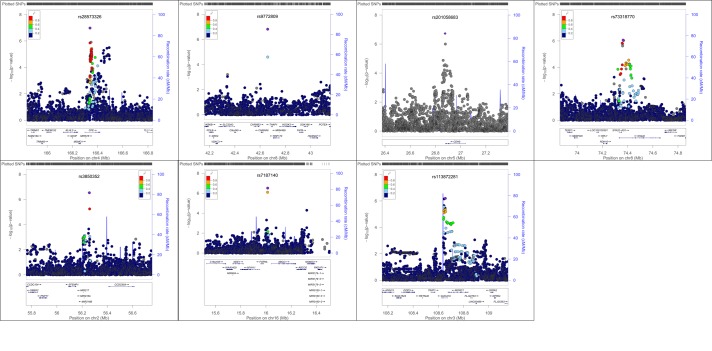
Regional association plots showing–log10(p-values) against base-pair position for African American (AA) women considering grade 0 vs. 1–3 analyses.

**Fig 5:**
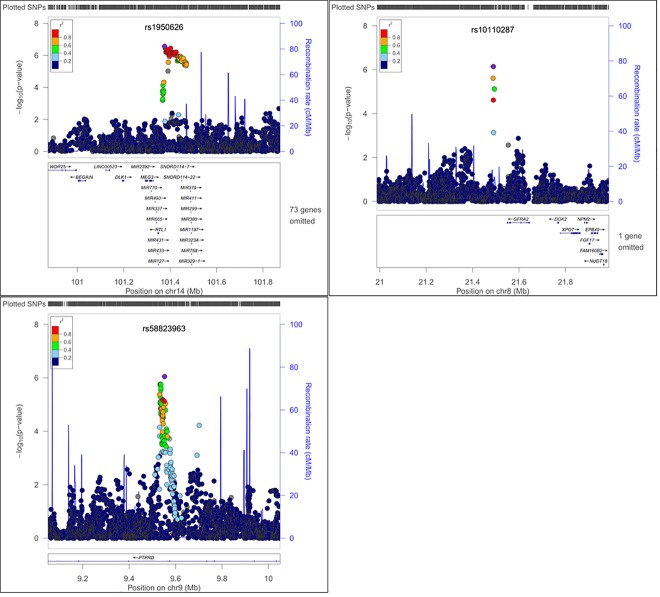
Regional association plots showing–log10(p-values) against base-pair position for Hispanic (HP) women considering grade 0 vs. 1–3 analyses.

In addition to evaluating women with any POP, we also analyzed AA and HP women with moderate-severe POP (grades 2–3), adjusting for the same variables described above (Table 3). Again, in race-ethic stratified analyses, no loci reached genome-wide significance, but we identified SNPs in seven loci in the AA population and SNPs in three loci in the HP population which were associated with grades 2–3 POP at a P value threshold of <1.00x10^-6^. In analyses specific to AA women, the most significant SNP associated with moderate/severe POP was SNP rs28573326 (OR: 2.14; 95% CI 1.62–2.83; P = 1.04x10^-7^) which is located in the intronic region of the carboxypeptidase E (*CPE*) gene. Similarly, in analyses specific to HP women we found several SNPs in and around the gene *AL132709*.*5* to be associated with grades 2–3 POP, with rs1950626 (OR: 2.96; 95% CI: 1.96–4.48; P = 2.64x10^-7^) as the most significant SNP in the region. Regional association plots for top loci from the grade 0 vs. 2–3 analyses are shown in Fig 4. for AA subgroup and Fig 5. for HP subgroup. Race/ethnicity specific correlation plots for top associated SNPs (P < 10^−5^) from analyses of either any POP or moderate/severe POP not only showed that the effect size estimates were in the same direction, but that the magnitude of the effect size was larger in the moderate/severe POP analyses than in the any POP analyses (S6 Fig).

**Table 3:** Top genetic loci associated with severe pelvic organ prolapse (grade 0 vs. 2–3) in African American (AA) and Hispanic (HP) women from the Women’s Health Initiative.

	SNP	CHR	BP	On/Nearby Gene	EA/RA	EAF	info	OR	95% CI	P
	rs28573326	4	166338328	*CPE* *	G/A	0.29	0.97	2.14	1.62–2.83	1.04x10^-7^
	rs9772809	8	42658181	*THAP1*,*CHRNA6*,*CHRNB3*	C/T	0.93	0.58	0.20	0.11–0.36	1.51x10^-7^
	rs201058683	5	26876667	*CDH9*	AT/A	0.21	0.79	2.47	1.76–3.47	1.88x10^-7^
**AA**	rs3850352	2	56255496	*EFEMP1*,*MIR216-A*,*B*,*MIR217*	C/T	0.82	0.95	0.42	0.31–0.59	2.91x10^-7^
	rs7187140	16	16011680	*FOPNL*,*ABCC1*,*MYH11*	C/T	0.09	0.43	5.35	2.82–10.16	3.02x10^-7^
	rs113872281	3	108653832	*GUCA1C* *,*MORC1*	G/T	0.17	0.98	2.27	1.65–3.14	6.27x10^-7^
	rs73318770	8	74362915	*STAU2* *	G/A	0.07	0.98	3.21	2.02–5.11	8.46x10^-7^
	rs1950626	14	101373973	*AL132709*.*5*,*MIR370*,*RTL1*	G/A	0.79	0.94	2.96	1.96–4.48	2.64x10^-7^
**HP**	rs10110287	8	21487130	*GFRA2*	T/C	0.08	0.78	3.69	2.20–6.18	7.05x10^-7^
	rs58823963	9	9550389	*PTPRD* *	A/C	0.12	0.98	2.69	1.81–3.99	8.81x10^-7^

CHR = Chromosome; SNP = Single Nucleotide Polymorphism; EA = Effect Allele; RA = Reference Allele

*SNP is on gene; EAF = Effect Allele Frequency for Controls; OR = Odds Ratio; CI = Confidence Interval; P = p-value from logistic regression; Logistic regression models were adjusted for age at ascertainment, BMI (continuous), parity continuous) and 4 genetic ancestry components (continuous)

We then aggregated the signals from the AA and HP analyses by performing inverse variance weighted fixed effects meta-analysis for both definitions of POP (Table 4): any POP (grades 1–3) and moderate-severe POP (grades 2–3). Fixed effects meta-analysis of AA and HP datasets for any severity of POP showed only one intronic SNP located in the *DPP6* gene to be associated with any POP at a P value threshold of <1x10^-6^ with similar effect sizes in both datasets: rs11243354 (Meta-analysis OR: 1.36; 5.22x10^-7^; I^2^ = 0). Meta-analysis of the AA and HP datasets for moderate-severe POP (grades 2–3) showed a SNP near the *PGBD5* gene (rs740494; OR: 2.17; P = 8.61x10^-7^) to be associated with POP. A more detailed list of SNPs from independent loci with P value threshold <10^−5^ for the any POP and moderate-severe POP analyses are shown in S2 Table and S3 Table.

**Table 4:** Meta-analysis of SNPs from race specific analyses using the Women’s Health Initiative.

	SNP	CHR	BP	Gene	EA/RA	AA				HP				Meta-analysis		
						EAF	Info	OR	P	EAF	Info	OR	P	OR	P	I^2^
**Any POP**	rs11243354	7	154379234	*DPP6* *	C/T	0.45	0.96	1.36	1.72x10^-05^	0.20	0.94	1.39	9.54x10^-03^	1.36	5.22x10^-7^	0
**POP**	rs740494	1	230644927	*PGBD5*	G/C	0.12	0.80	2.29	8.60x10^-05^	0.12	0.80	2.02	2.77x10^-03^	2.17	8.61x10^-7^	0
**Grades 2–3**	rs2209875	9	91813461	*SHC3*	A/G	0.31	0.99	0.64	1.44x10^-03^	0.48	0.99	0.55	1.40x10^-04^	0.60	9.28x10^-7^	0

CHR = Chromosome; SNP = Single Nucleotide Polymorphism; EA = Effect Allele; RA = Reference Allele

*SNP is on gene; EAF = Effect Allele Frequency for Controls; OR = Odds Ratio; CI = Confidence Interval; AA = African American; HP = Hispanic; Logistic regression models were adjusted for age at ascertainment, BMI (continuous), parity continuous) and 4 genetic ancestry components (continuous)

To evaluate the robustness of SNPs with the lowest P values in our study (P<10^−6^), we performed several sensitivity analyses. To evaluate if there was more than one independent SNP contributing to the signals in a given region, we regressed SNPs in the region while conditioning on the SNP with the smallest P value for that respective region. We did not identify any additional SNPs that had signals <10^−6^ after conditional analyses. Furthermore, since women who had previously had hysterectomy at baseline assessment were less likely to have/develop POP, we reevaluated the top SNPs (P<10^−5^) from our original models while additionally adjusting for hysterectomy status (S7 Fig). The effect estimates we observed from these models were comparable to the original models. Correlation plots for analyses with and without adjustment for hysterectomy for top hits are shown in S3 Fig. Additionally, performing analyses for the top SNPs by strata of hysterectomy status provided comparable results with high R^2^ estimates (S8 Fig). Finally, we provide effect estimates for six previously reported genome-wide significant SNPs [19]. We found suggestive evidence for rs1810636 (OR: 1.29; 95% CI: 0.99–1.69; P = 0.06) in our AA analyses when analyzing grade 0 vs. grades 2–3 POP. The magnitude and direction of OR for other SNPs were not consistent across ethnicities and close to the null (Table 5).

**Table 5:** ORs and minor allele frequencies of six statistically significant SNPs evaluated by *Allen-Brady et al. in current analyses evaluating Grade 0 vs. 2–3 POP analyses using data from the Women’s Health Initiative.

			WHI-AA Grade 0 vs. 2–3 Analysis			WHI-HP Grade 0 vs. 2–3 Analysis			1000G Phase 3 Samples**
Marker	Location	Gene	*P*	OR	MAF	*P*	OR	MAF	MAF
** **	** **	** **	** **	** **	**Controls**	** **	** **	**Controls**	**CEU/YRI/ASW/MXL**
rs1455311	4q21.21	Intergenic	0.94	1.02	0.08	0.97	0.99	0.12	0.19/0.03/0.11/0.09
rs1036819	8q24.22	*ZFAT*	0.62	0.88	0.07	0.39	1.22	0.11	0.12/0.06/0.02/0.16
rs430794	9q22.2	Intergenic	0.49	1.09	0.46	0.27	1.18	0.36	0.28/0.49/0.45/0.38
rs8027714	15q11.2	Intergenic	0.35	0.87	0.28	NA	NA	NA	0.04/0.31/0.29/0.02
rs1810636	20p13	Intergenic	0.06	1.29	0.29	0.14	0.80	0.38	0.34/0.26/0.30/0.22
rs2236479	21q22.3	*COL18A1*	0.99	0.99	0.49	0.74	1.05	0.38	0.32/0.53/0.48/0.34

MAF = Minor allele frequency; OR = odds ratio; 1000G = 1000 Genomes Project; NA = Not applicable as SNP was not assessed due to low minor allele frequency

*Refers to SNPs published in Table 2 of article by Allen-Brady et al. [19]

**Source for reference population minor allele frequencies: http://www.ncbi.nlm.nih.gov/variation/tools/1000genomes/

## Discussion

We observed several potential loci which may be associated with POP among AA and HP women in the Women’s Health Initiative. While strong associations were observed, these associations were not genome-wide significant. However, we note that several of the associations were consistent with regards to the direction and magnitude across populations. Additionally, the magnitudes of associations were generally larger for the grade 0 vs 2–3 analyses than for grade 0 vs. 1–3 analyses. This is consistent with the hypothesis that women with a strong genetic risk for POP are more likely to have advanced POP. The strongest observed associations (smallest p-values) for grade 0 vs 2&3 were in both AAs (*CPE* gene) and HPs (nearby *AL132709*.*5*, *MIR370* and *RTL1*). Meta-analysis of grade 0 vs. 1–3 analysis identified a SNP in the gene *DPP6* had the strongest association with POP.

*CPE* is a secretory protein involved in the biosynthesis of peptide hormones and neurotransmitters, such as insulin, involved in energy balance, nutrient partition, and satiety [33]. Animal model studies have associated mutations in the *CPE* gene with obesity, diabetes, infertility, and hyperinsulinemia in mice [34;35]. Although, there is no known direct relationship between *CPE* and POP, high BMI is an important risk factor for POP. Furthermore, metabolic syndrome, which involves obesity, cardiac risk factors, insulin resistance, systemic inflammation, atherogenic dyslipidemia and endothelial dysfunction, has associated with multiple urologic conditions including POP [36]. The strongest associated variant in HPs was not in a gene, but was located near *AL132709*.*5*, *MIR370*, and *RTL1*. There is no established relationship between these genes and POP or POP-related phenotypes. Another locus surrounding SNP rs7187140, identified in the AA grade 0 vs. 2–3 analysis, is also noteworthy in relation to POP. Although the SNP is intergenic, it is within 60 kb of the smooth muscle myosin heavy chain 11 (*MYH11*) gene. In a small gene expression study in tissue from anterior vaginal walls, Bortolini and colleagues showed *MYH11* gene expression was down regulated in pre-menopausal women with POP compared with premenopausal women without POP [37].

Meta-analyses identified strong associations on the gene *DPP6*. The direction of the association for the most statistically significant SNP (rs11273354) was consistent across AAs and HPs. The SNP was also common in both populations (minor allele frequency >0.05). *DPP6* is a single transmembrane protein an important protein for voltage-gated ion channels in determining cellular excitability. To date, there have not been any reports of an association of *DPP6* with POP or any POP-related phenotypes. It is of interest that base-pair variation in the SNP rs11273354 alters binding motif of transcription factors containing the forkhead box D1 (*FOXD1*) gene [38]. Evaluation of expression quantitative trait loci (eQTL) using the GTEx portal [39] showed several SNPs in the *DPP6* gene were associated with subcutaneous adiposity, potentially suggesting indirect ties between obesity and POP which is not fully understood. *DPP6* has been associated with pancreatic cancer, tardive dyskinesia and amyotrophic lateral sclerosis in prior studies [40–44]. The piggyBac transposable element derived 5 (*PGBD5*) gene, identified in the grade 0 vs. 2–3 meta-analysis may also be a potential gene of interest in relation to POP. Although there have been no direct ties between *PGBD5* and POP in the literature, it is of interest that several eQTL have been shown to alter expression of this gene in the tibial nerve [39]. The tibial nerve originates from the same lumbar and sacral nerves (L5 through S3) which also innervate the pelvic floor [45]. Studies suggest that neuro-modulation through percutaneous or posterior tibial nerve stimulation, may be beneficial in alleviating symptoms associated with pelvic floor disorders including fecal and urinary incontinence [46–48].

We took several measures to ensure the validity of the observed associations. In the WHI-HT a large proportion of women had undergone hysterectomy prior to study enrollment, with no information on the indication for hysterectomy. Considering that POP is the 3^rd^ leading indication for hysterectomy in women, it is then possible that some women might have had POP previously but was not detected at baseline. At the same time, women without a uterus can still develop other forms of prolapse such as rectocele and cystocele, as was the case during follow-up. To address this concern we performed analyses adjusting for hysterectomy and by strata of hysterectomy for our top hits and showed that these estimates were highly correlated. Therefore, bias introduced by hysterectomy status is not a likely explanation for associations observed in this study. Additionally, we compared the magnitudes of associations for our top hits in the any POP and moderate-severe POP analyses to show that not only were these associations in the same direction, but that the magnitude of effect estimates were larger for analyses considering moderate-severe POP than any POP. Effect estimates in the any POP analyses were likely driven by moderate-severe POP cases. One additional advantage of using the WHI-HT dataset is that information on important risk factors for POP was collected uniformly across all patients. In our analyses we adjusted for age, BMI and parity as these have been shown to be important risk factors for POP. Ideally, adjustment for mode of delivery would have provided for a more robust analysis, however, the WHI-HT did not collect this information.

We were not able to replicate the associations observed in a previously published GWAS by Allen-Brady et al., in a EA population with 115 cases and 2,976 controls [19]. Although cases were well-characterized, the study utilized general-population controls for whom the phenotype status was not verified. The study identified associations in six chromosomal regions 4q21, 8q24, 9q22, 15q11, 20p13, and 21q22. Two of the strongest associated SNPs were located within a gene: rs2236479 (*COL18A1*) and rs1036819 (*ZFAT*). The odds ratios we observed for these loci were close to the null and corresponding P values were >0.05. Potential reasons we were unable to replicate these findings are that genetic variants for POP may differ across racial/ethnic populations or power, as the minor allele frequency for these previously associated variants varies significantly across geographic populations.

To our knowledge, this is the second GWAS that evaluates POP risk and the first GWAS in AA and HP women. Majority of other genetic investigations evaluating POP risk have been limited to relatively smaller candidate gene studies that primarily focused on women of European or Asian descent [21]. The lack of GWAS evaluating POP, especially in minority populations and the lack of replication of candidate gene signals across existing studies are likely due to several factors and barriers, as highlighted by Wu et al. [49]. First, POP is a difficult phenotype to characterize as it involves a specialized pelvic exam and is rarely evaluated in research studies as routine practice. Secondly, a well conducted genetic epidemiologic study also equally rests on proper characterization of controls to reduce outcome misclassification. Prevalence of POP increases with age, especially after menopause and is highly prevalent in this population. The timing of control classification is thus of crucial importance, with ideal preference for including older post-menopausal women with no POP over younger pre-menopausal women to potentially reduce outcome misclassification.

Using the WHI-HT data allowed us to overcome these two barriers to a certain degree to conduct the study at hand. The WHI-HT used centrally validated standardized procedures performed by trained medical staff to collect information on POP at baseline and at up to 10-annual visits per individual (all of whom were post-menopausal). This not only allowed us to properly identify POP cases who developed POP during follow-up visits, but also allowed us to classify women as controls considering multiple visits, when possible. Even though is a marked improvement over previous studies that have investigated POP, there is likely some degree of misclassification in our study as well. While information on POP was available for multiple visits, the loss-to-follow-up rate in the WHI-HT was extremely high. Consequently, for controls identified at baseline that were lost to follow-up, we could not determine POP status through time. For majority of the cases who had two or more assessments of POP (612 AA POP cases and 498 HP POP cases), POP could be verified at two or more visits for 70% of these AA POP cases and 80% of these HP POP cases. For some women POP grade fluctuated between visits; this speaks to a degree of subjectivity in POP measurement even when using standardized procedures. For women with information on POP for multiple visits, we noted the visit at the first ascertainment of POP when considering any POP and the first ascertainment of grade 2 or higher POP when considering moderate/severe POP. It should also be noted that POP was measured in the supine lithotomy position in the WHI-HT, and therefore, the maximal extant of prolapse was likely underestimated compared with a semi-upright or upright position of assessment, which is ideal for determining the maximum extent of prolapse.

Thirdly, resources for large scale genetic investigations for phenotypes such as POP are extremely limited, making replication of findings a difficult task to achieve. This especially rings true for investigation in minority populations such as AAs and HPs, who have historically been under-represented in medical research in general. We were unable to replicate our findings in an independent cohort of AA and HP women, and stress the need for an independent replication of our findings. To address this, we performed trans-ethnic replication across AAs and HPs. Although we have a strong sample size of 2,701 women, the number of women with POP was limited (any POP: n = 1,382). However, a small proportion of women (n = 317) had grades 2–3 POP and even fewer women had POP which protruded beyond the introitus. This resulted in our analysis being underpowered to detect smaller associations. Given the highly stringent p-value threshold due to multiple testing, the possibility that our findings are due to chance alone cannot be ruled out. Additionally, since we had only two datasets for meta-analysis, we resorted to using a fixed effects meta-analysis framework, instead of newer trans-ethnic meta-analysis techniques such as new-random-effects model [31] or MANTRA [32]. Both these methods have been shown to boost power especially in scenarios with allele frequency differences and/or extreme allelic heterogeneity [50]. Simulations of a considerably larger number of studies (N = 30) have demonstrated considerably improved power for these techniques, however, these techniques are also underpowered as the number of meta-analysis studies decrease (N = 10) in the presence of effect-size heterogeneity [50]. The added benefit from utilizing these techniques when the number of studies is small (N = 2) is not clear. By design our meta-analysis is most sensitive to identifying associations for variants with similar allele frequencies and effect estimates across populations. Considering lower prevalence of POP in AA women compared with HP women it is likely that there may be ancestry specific loci which we were not able to detect through this meta-analytic technique. Future meta-analysis of a greater number of studies with diverse populations, coupled with meta-analysis techniques designed to leverage heterogeneity associated with between population differences may provide insight into these loci.

To our knowledge, the WHI-HT data is the only resource available which has validated detailed information on POP and simultaneous availability of genetic data. The surge of interest in utilizing Electronic medical record (EMR) databases for research investigation provides future hope for replicating these results. However, constructing a well characterized set of cases and controls requires building algorithms that can identify cases and controls with a high degree of sensitivity and specificity, respectively. To this end, our group is currently in the process of developing algorithms using EMR data from the BioVU DNA repository at Vanderbilt University, which we hope to apply in larger EMR database networks with available GWAS data for replication in the future.

Little is known about POP pathophysiology or genetic risk factors, particularly among AA and HP women. Our study is the first POP GWAS in minority women. Our findings suggest that common germ-line variations may contribute to increased risk for POP among AAs and HPs; however, further research with larger minority sample sizes is necessary to validate our study findings.

## Supporting Information

S1 Fig Multidimensional Scaling (MDS) Components plot of WHI POP cases and controls compared with HAPMAP reference e populations. MDS axes for African Americans and Hispanics were plotted with MDS1 on the y-axis and MDS2 on the x-axis. Values were color coded according to self-reported race among cases and controls and compared to HAPMAP reference populations. Populations are labeled in the legend within the figure.
(TIF)

S2 Fig Manhattan plot of single SNP association analyses considering grade 0 vs. grade 1–3 POP for African American (AA) women from the Women’s Health Initiative. Logistic regression models were adjusted for age at ascertainment (continuous), body mass index (continuous), parity (continuous) and genetic ancestry components (continuous). X-axis: base-pair position; Y-axis: -log10(p-values).
(TIF)

S3 Fig Manhattan plot of single SNP association analyses considering grade 0 vs. grade 1–3 POP for Hispanic (HP) women from the Women’s Health Initiative. Logistic regression models were adjusted for age at ascertainment (continuous), body mass index (continuous), parity (continuous) and genetic ancestry components (continuous). X-axis: base-pair position; Y-axis: -log10(p-values).
(TIF)

S4 Fig Manhattan plot of single SNP association analyses considering grade 0 vs. grade 2–3 POP for African American (AA) women from the Women’s Health Initiative. Logistic regression models were adjusted for age at ascertainment (continuous), body mass index (continuous), parity (continuous) and genetic ancestry components (continuous). X-axis: base-pair position; Y-axis: -log10(p-values).
(TIF)

S5 Fig Manhattan plot of single SNP association analyses considering grade 0 vs. grade 2–3 POP for Hispanic (HP) women from the Women’s Health Initiative. Logistic regression models were adjusted for age at ascertainment (continuous), body mass index (continuous), parity (continuous) and genetic ancestry components (continuous). X-axis: base-pair position; Y-axis: -log10(p-values).
(TIF)

S6 Fig Compares log(OR) estimates from single SNP association analyses from grade 0 vs. any POP models to grade 0 vs. moderate/severe POP models. This figure compares beta-estimates of top hits (p <10–5) originating from either any POP or moderate/severe POP models where all models were adjusted for age, BMI, parity and ancestry components. X-axis and Y-axis represents natural log transformed odds ratios from any POP and moderate/severe POP models, respectively. left plot: AAs; right plot: HPs.
(TIF)

S7 Fig Summary of log(OR) of single SNP association analyses adjusted for hysterectomy compared to no adjustment for hysterectomy. This figure compares beta-estimates of top hits (p < 10^−5^) from models adjusted for age, BMI, parity and ancestry components to models adjusted for hysterectomy in addition to the aforementioned variables. Original refers to betas from models adjusted for age, BMI, parity and ancestry components; Hysterectomy adjusted refers to betas from models adjusted for hysterectomy status in addition to factors in the original models.
(TIF)

S8 Fig Summary of log(OR) estimates from single SNP association analyses by strata of hysterectomy status. This figure compares beta-estimates of top hits (p < 10^−5^) from models adjusted for age, BMI, parity and ancestry components in women with and without a hysterectomy at baseline. X-axis and Y-axis represents natural log transformed odds ratios from analyses in women without hysterectomy and women with hysterectomy, respectively. Top 2 plots are for AAs (Any POP and moderate/severe POP, left to right). Bottom two plots are for HPs (Any POP and moderate/severe POP, left to right).
(TIF)

S1 Table Associations between key risk factors for any pelvic organ prolapse and grades 2–3 pelvic organ prolapse in the WHI Hormone Therapy trial. The following table presents the associations between key risk factors in relation to any POP (grades 1–3) and severe/moderate POP (grades 2–3).
(DOCX)

S2 Table Results from meta-analysis of Grade 0 vs. Grade 1–3 POP across African American (AA) and Hispanic (HP) Women from the Women’s Health Initiative. The following table presents results on single nucleotide polymorphisms (SNPs) associated with Grade 1–3 prolapse, which had p < 0.00001 in the fixed effects meta-analysis. Results from random-effects models and Heterogeneity Scores (I^2^) are also presented.
(DOCX)

S3 Table Results from meta-analysis of Grade 0 vs. Grade 2–3 POP across African American (AA) and Hispanic (HP) women from the Women’s Health Initiative. The following table presents results on single nucleotide polymorphisms (SNPs) associated with Grade 2–3 prolapse, which had p < 0.00001 in the fixed effects meta-analysis. Results from random-effects models and Heterogeneity Scores (I^2^) are also presented.
(DOCX)

## References

[1] Hendrix SL, Clark A, Nygaard I, Aragaki A, Barnabei V, McTiernan A. Pelvic organ prolapse in the Women's Health Initiative: gravity and gravidity. Am J Obstet Gynecol 2002 6;186(6):1160–6. 12066091 10.1067/mob.2002.123819

[2] Wu JM, Matthews CA, Conover MM, Pate V, Funk MJ. Lifetime risk of stress urinary incontinence or pelvic organ prolapse surgery. Obstetrics & Gynecology 2014;123(6):1201–6. 10.1097/AOG.0000000000000286 24807341 PMC4174312

[3] Nygaard I, Barber MD, Burgio KL, Kenton K, Meikle S, Schaffer J, et al Prevalence of symptomatic pelvic floor disorders in US women. JAMA 2008 9 17;300(11):1311–6. 10.1001/jama.300.11.1311 18799443 PMC2918416

[4] Whitcomb EL, Rortveit G, Brown JS, Creasman JM, Thom DH, Van Den Eeden SK, et al Racial differences in pelvic organ prolapse. Obstet Gynecol 2009 12;114(6):1271–7. 10.1097/AOG.0b013e3181bf9cc8 19935029 PMC2879888

[5] Rortveit G, Brown JS, Thom DH, Van Den Eeden SK, Creasman JM, Subak LL. Symptomatic Pelvic Organ Prolapse: Prevalence and Risk Factors in a Population-Based, Racially Diverse Cohort. Obstetrics & Gynecology 2007;109(6). 10.1097/01.AOG.0000263469.68106.90 17540813

[6] Glazener C, Elders A, MacArthur C, Lancashire RJ, Herbison P, Hagen S, et al Childbirth and prolapse: long-term associations with the symptoms and objective measurement of pelvic organ prolapse. BJOG: An International Journal of Obstetrics & Gynaecology 2013;120(2):161–8. 10.1111/1471-0528.12075 23190018

[7] Bradley CS, Zimmerman MB, Qi Y, Nygaard IE. Natural history of pelvic organ prolapse in postmenopausal women. Obstet Gynecol 2007 4;109(4):848–54. 17400845 10.1097/01.AOG.0000255977.91296.5d

[8] Kudish BI, Iglesia CB, Sokol RJ, Cochrane B, Richter HE, Larson J, et al Effect of weight change on natural history of pelvic organ prolapse. Obstet Gynecol 2009 1;113(1):81–8. 10.1097/AOG.0b013e318190a0dd 19104363 PMC2684063

[9] Buchsbaum GM, Duecy EE, Kerr LA, Huang LS, Perevich M, Guzick DS. Pelvic organ prolapse in nulliparous women and their parous sisters. Obstet Gynecol 2006 12;108(6):1388–93. 17138771 10.1097/01.AOG.0000245784.31082.ed

[10] Chiaffarino F, Chatenoud L, Dindelli M, Meschia M, Buonaguidi A, Amicarelli F, et al Reproductive factors, family history, occupation and risk of urogenital prolapse. Eur J Obstet Gynecol Reprod Biol 1999 1;82(1):63–7. 10192487 10.1016/s0301-2115(98)00175-4

[11] McLennan MT, Harris JK, Kariuki B, Meyer S. Family history as a risk factor for pelvic organ prolapse. Int Urogynecol J Pelvic Floor Dysfunct 2008 8;19(8):1063–9. 10.1007/s00192-008-0591-1 18350241

[12] Nikolova G, Lee H, Berkovitz S, Nelson S, Sinsheimer J, Vilain E, et al Sequence variant in the laminin gamma1 (LAMC1) gene associated with familial pelvic organ prolapse. Hum Genet 2007 2;120(6):847–56. 17021862 10.1007/s00439-006-0267-1

[13] Norton PA, Allen-Brady K, Cannon-Albright LA. The familiality of pelvic organ prolapse in the Utah Population Database. Int Urogynecol J 2013 Mar;24(3):413–8. 10.1007/s00192-012-1866-0 22890278

[14] Chen C, Hill LD, Schubert CM, Strauss JF III, Matthews CA. Is laminin gamma-1 a candidate gene for advanced pelvic organ prolapse? Am J Obstet Gynecol 2010 5;202(5):505 10.1016/j.ajog.2010.01.014 20223449 PMC2866811

[15] Chen HY, Lin WY, Chen YH, Chen WC, Tsai FJ, Tsai CH. Matrix metalloproteinase-9 polymorphism and risk of pelvic organ prolapse in Taiwanese women. Eur J Obstet Gynecol Reprod Biol 2010 4;149(2):222–4. 10.1016/j.ejogrb.2009.12.014 20144500

[16] Kluivers KB, Dijkstra JR, Hendriks JC, Lince SL, Vierhout ME, van Kempen LC. COL3A1 2209G>A is a predictor of pelvic organ prolapse. Int Urogynecol J Pelvic Floor Dysfunct 2009 9;20(9):1113–8. 10.1007/s00192-009-0913-y 19444361

[17] Wu JM, Visco AG, Grass EA, Craig DM, Fulton RG, Haynes C, et al Comprehensive analysis of LAMC1 genetic variants in advanced pelvic organ prolapse. Am J Obstet Gynecol 2012 5;206(5):447–6. 10.1016/j.ajog.2012.01.033 22342894 PMC3508006

[18] Wu JM, Visco AG, Grass EA, Craig DM, Fulton RG, Haynes C, et al Matrix Metalloproteinase-9 Genetic Polymorphisms and the Risk for Advanced Pelvic Organ Prolapse. Obstetrics & Gynecology 2012;120(3):587–93. 10.1097/AOG.0b013e318262234b 22914468 PMC3427536

[19] Allen-Brady K, Cannon-Albright L, Farnham JM, Teerlink C, Vierhout ME, van Kempen LoC, et al Identification of six loci associated with pelvic organ prolapse using genome-wide association analysis. Obstetrics and gynecology 2011;118(6):1345 10.1097/AOG.0b013e318236f4b5 22105264 PMC3233378

[20] Allen-Brady K, Cannon-Albright LA, Farnham JM, Norton PA. Evidence for Pelvic Organ Prolapse Predisposition Genes on Chromosomes 10 and 17. American Journal of Obstetrics and Gynecology 2014. 10.1016/j.ajog.2014.12.037 PMC4457667 25557205

[21] Ward RM, Edwards DRV, Edwards T, Giri A, Jerome RN, Wu JM. Genetic epidemiology of pelvic organ prolapse: a systematic review. American Journal of Obstetrics and Gynecology 2014;211(4):326–35. 10.1016/j.ajog.2014.04.006 24721264 PMC4213176

[22] Women's Health Initiative Study Group. Design of the Women's Health Initiative Clinical Trial and Observational Study-examples from the Women's Health Initiative. Controlled clinical trials 1998;19(1):61–109. 9492970 10.1016/s0197-2456(97)00078-0

[23] Curb JD, McTiernan A, Heckbert SR, Kooperberg C, Stanford J, Nevitt M, et al Outcomes ascertainment and adjudication methods in the Women's Health Initiative. Ann Epidemiol 2003 10;13(9 Suppl):S122–S128. 14575944 10.1016/s1047-2797(03)00048-6

[24] Hays J, Hunt JR, Hubbell FA, Anderson GL, Limacher M, Allen C, et al The Women's Health Initiative recruitment methods and results. Ann Epidemiol 2003 10;13(9 Suppl):S18–S77. 14575939 10.1016/s1047-2797(03)00042-5

[25] Korn JM, Kuruvilla FG, McCarroll SA, Wysoker A, Nemesh J, Cawley S, et al Integrated genotype calling and association analysis of SNPs, common copy number polymorphisms and rare CNVs. Nat Genet 2008 10;40(10):1253–60. 10.1038/ng.237 18776909 PMC2756534

[26] Purcell S, Neale B, Todd-Brown K, Thomas L, Ferreira MA, Bender D, et al PLINK: a tool set for whole-genome association and population-based linkage analyses. The American Journal of Human Genetics 2007;81(3):559–75. 17701901 10.1086/519795 PMC1950838

[27] Howie BN, Donnelly P, Marchini J. A flexible and accurate genotype imputation method for the next generation of genome-wide association studies. PLoS Genet 2009 6;5(6):e1000529 10.1371/journal.pgen.1000529 19543373 PMC2689936

[28] Howie B, Marchini J, Stephens M. Genotype imputation with thousands of genomes. G3: Genes, Genomes, Genetics 2011;1(6):457–70. 22384356 10.1534/g3.111.001198 PMC3276165

[29] Delaneau O, Marchini J, Zagury JF. A linear complexity phasing method for thousands of genomes. Nat Methods 2012 2;9(2):179–81. 10.1038/nmeth.1785 22138821

[30] Marchini J, Howie B, Myers S, McVean G, Donnelly P. A new multipoint method for genome-wide association studies by imputation of genotypes. Nat Genet 2007 7;39(7):906–13. 17572673 10.1038/ng2088

[31] Han B, Eskin E. Random-effects model aimed at discovering associations in meta-analysis of genome-wide association studies. The American Journal of Human Genetics 2011;88(5):586–98. 10.1016/j.ajhg.2011.04.014 21565292 PMC3146723

[32] Morris AP. Transethnic meta-analysis of genomewide association studies. Genetic epidemiology 2011;35(8):809–22. 10.1002/gepi.20630 22125221 PMC3460225

[33] Chen H, Jawahar S, Qian Y, Duong Q, Chan G, Parker A, et al Missense polymorphism in the human carboxypeptidase E gene alters enzymatic activity. Hum Mutat 2001 8;18(2):120–31. 11462236 10.1002/humu.1161

[34] Jackson RS, Creemers JW, Ohagi S, Raffin-Sanson ML, Sanders L, Montague CT, et al Obesity and impaired prohormone processing associated with mutations in the human prohormone convertase 1 gene. Nat Genet 1997 7;16(3):303–6. 9207799 10.1038/ng0797-303

[35] Naggert JK, Fricker LD, Varlamov O, Nishina PM, Rouille Y, Steiner DF, et al Hyperproinsulinaemia in obese fat/fat mice associated with a carboxypeptidase E mutation which reduces enzyme activity. Nat Genet 1995 6;10(2):135–42. 7663508 10.1038/ng0695-135

[36] Chu KF, Rotker K, Ellsworth P. The impact of obesity on benign and malignant urologic conditions. Postgraduate medicine 2013;125(4):53–69. 10.3810/pgm.2013.07.2679 23933894

[37] Bortolini MA, Shynlova O, Drutz HP, Castro RA, Girão MJ, Lye S, et al Expression of genes encoding smooth muscle contractile proteins in vaginal tissue of women with and without pelvic organ prolapse. Neurourology and urodynamics 2012;31(1):109–14. 10.1002/nau.21175 22038928

[38] Ward LD, Kellis M. HaploReg: a resource for exploring chromatin states, conservation, and regulatory motif alterations within sets of genetically linked variants. Nucleic acids research 2012;40(D1):D930–D934. 22064851 10.1093/nar/gkr917 PMC3245002

[39] Lonsdale J, Thomas J, Salvatore M, Phillips R, Lo E, Shad S, et al The genotype-tissue expression (GTEx) project. Nature genetics 2013;45(6):580–5. 10.1038/ng.2653 23715323 PMC4010069

[40] Tanaka S, Syu A, Ishiguro H, Inada T, Horiuchi Y, Ishikawa M, et al DPP6 as a candidate gene for neuroleptic-induced tardive dyskinesia. Pharmacogenomics J 2013 2;13(1):27–34. 10.1038/tpj.2011.36 21826085

[41] Low SK, Kuchiba A, Zembutsu H, Saito A, Takahashi A, Kubo M, et al Genome-wide association study of pancreatic cancer in Japanese population. PLoS One 2010;5(7):e11824 10.1371/journal.pone.0011824 20686608 PMC2912284

[42] Cronin S, Tomik B, Bradley DG, Slowik A, Hardiman O. Screening for replication of genome-wide SNP associations in sporadic ALS. Eur J Hum Genet 2009 2;17(2):213–8. 10.1038/ejhg.2008.194 18987618 PMC2986065

[43] van Es MA, van Vught PW, Blauw HM, Franke L, Saris CG, Van den Bosch L, et al Genetic variation in DPP6 is associated with susceptibility to amyotrophic lateral sclerosis. Nat Genet 2008 1;40(1):29–31. 18084291 10.1038/ng.2007.52

[44] Cronin S, Berger S, Ding J, Schymick JC, Washecka N, Hernandez DG, et al A genome-wide association study of sporadic ALS in a homogenous Irish population. Hum Mol Genet 2008 3 1;17(5):768–74. 18057069 10.1093/hmg/ddm361

[45] Marani E, Koch WF. Innervation of the Mature Human Pelvis The Pelvis. Springer; 2014 p. 337–59.

[46] Edenfield AL, Amundsen CL, Wu JM, Levin PJ, Siddiqui NY. Posterior Tibial Nerve Stimulation for the Treatment of Fecal Incontinence: A Systematic Evidence Review. Obstetrical & gynecological survey 2015;70(5):329–41. 10.1097/OGX.0000000000000171 25974730

[47] van Balken MR, Vandoninck V, Gisolf KW, Vergunst H, Kiemeney LA, Debruyne FM, et al Posterior tibial nerve stimulation as neuromodulative treatment of lower urinary tract dysfunction. The Journal of urology 2001;166(3):914–8. 11490245 10.1097/00005392-200109000-00025

[48] Biemans JM, van Balken MR. Efficacy and effectiveness of percutaneous tibial nerve stimulation in the treatment of pelvic organ disorders: a systematic review. Neuromodulation: Technology at the Neural Interface 2013;16(1):25–34. 10.1111/j.1525-1403.2012.00504.x 22985128

[49] Wu JM, Ward ReM, Allen-Brady KL, Edwards TL, Norton PA, Hartmann KE, et al Phenotyping clinical disorders: lessons learned from pelvic organ prolapse. American Journal of Obstetrics and Gynecology 2013;208(5):360–5. 10.1016/j.ajog.2012.11.030 23200709 PMC3597745

[50] Wang X, Chua HX, Chen P, Ong RT-H, Sim X, Zhang W, et al Comparing methods for performing trans-ethnic meta-analysis of genome-wide association studies. Human molecular genetics 2013;ddt064. 10.1093/hmg/ddt064 23406875

