# Supplementary figures and images for: Genetic Determinants of Pelvic Organ Prolapse among African American and Hispanic Women in the Women’s Health Initiative
Source: PLoS One. 2015 Nov 6;10(11):e0141647. doi: 10.1371/journal.pone.0141647 (PMC4636147; doi:10.1371/journal.pone.0141647)

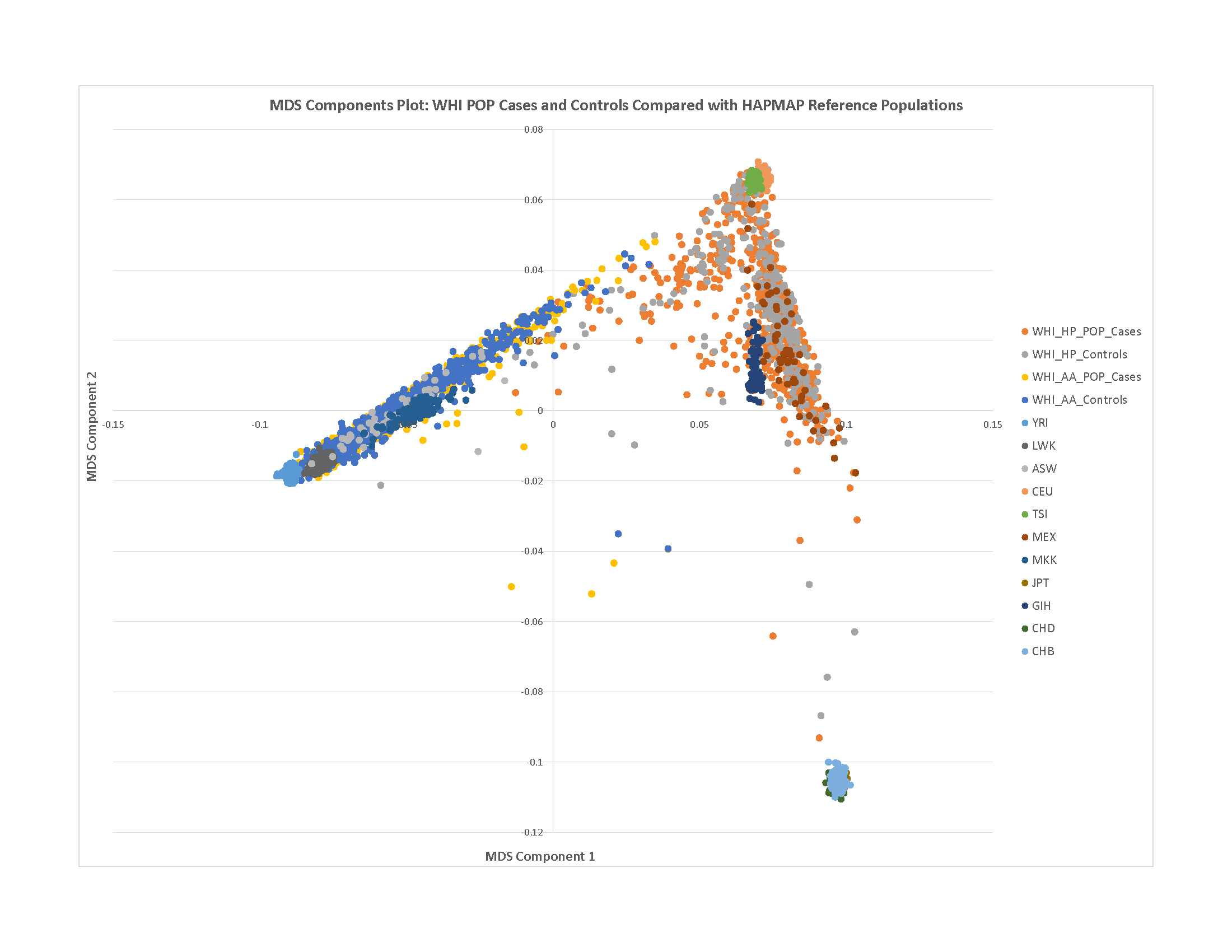

Supplement: S1 Fig — MDS axes for African Americans and Hispanics were plotted with MDS1 on the y-axis and MDS2 on the x-axis. Values were color coded according to self-reported race among cases and controls and compared to HAPMAP reference populations. Populations are labeled in the legend within the figure. (TIF) [file pone.0141647.s001.tif]

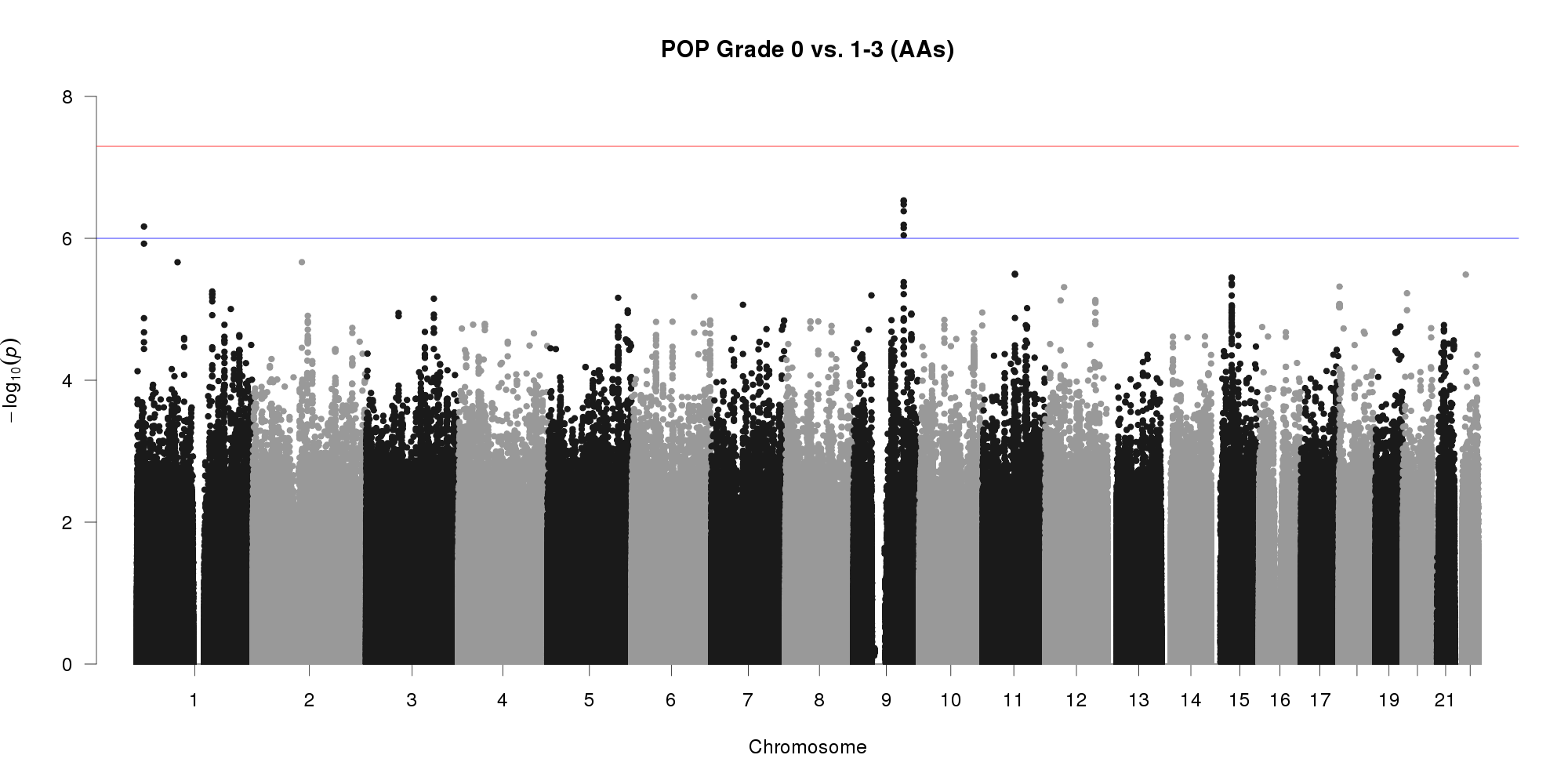

Supplement: S2 Fig — Logistic regression models were adjusted for age at ascertainment (continuous), body mass index (continuous), parity (continuous) and genetic ancestry components (continuous). X-axis: base-pair position; Y-axis: -log10(p-values). (TIF) [file pone.0141647.s002.tif]

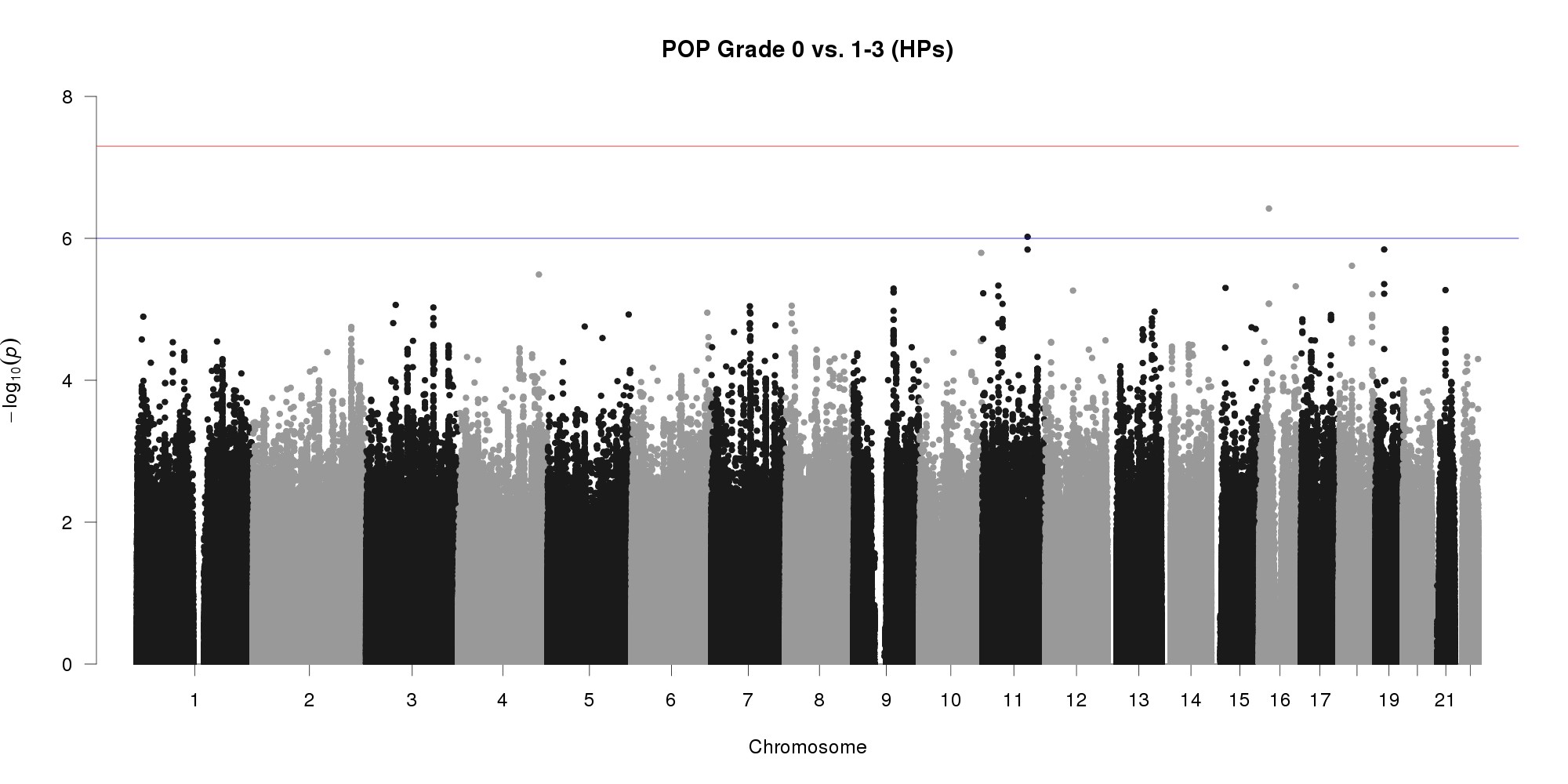

Supplement: S3 Fig — Logistic regression models were adjusted for age at ascertainment (continuous), body mass index (continuous), parity (continuous) and genetic ancestry components (continuous). X-axis: base-pair position; Y-axis: -log10(p-values). (TIF) [file pone.0141647.s003.tif]

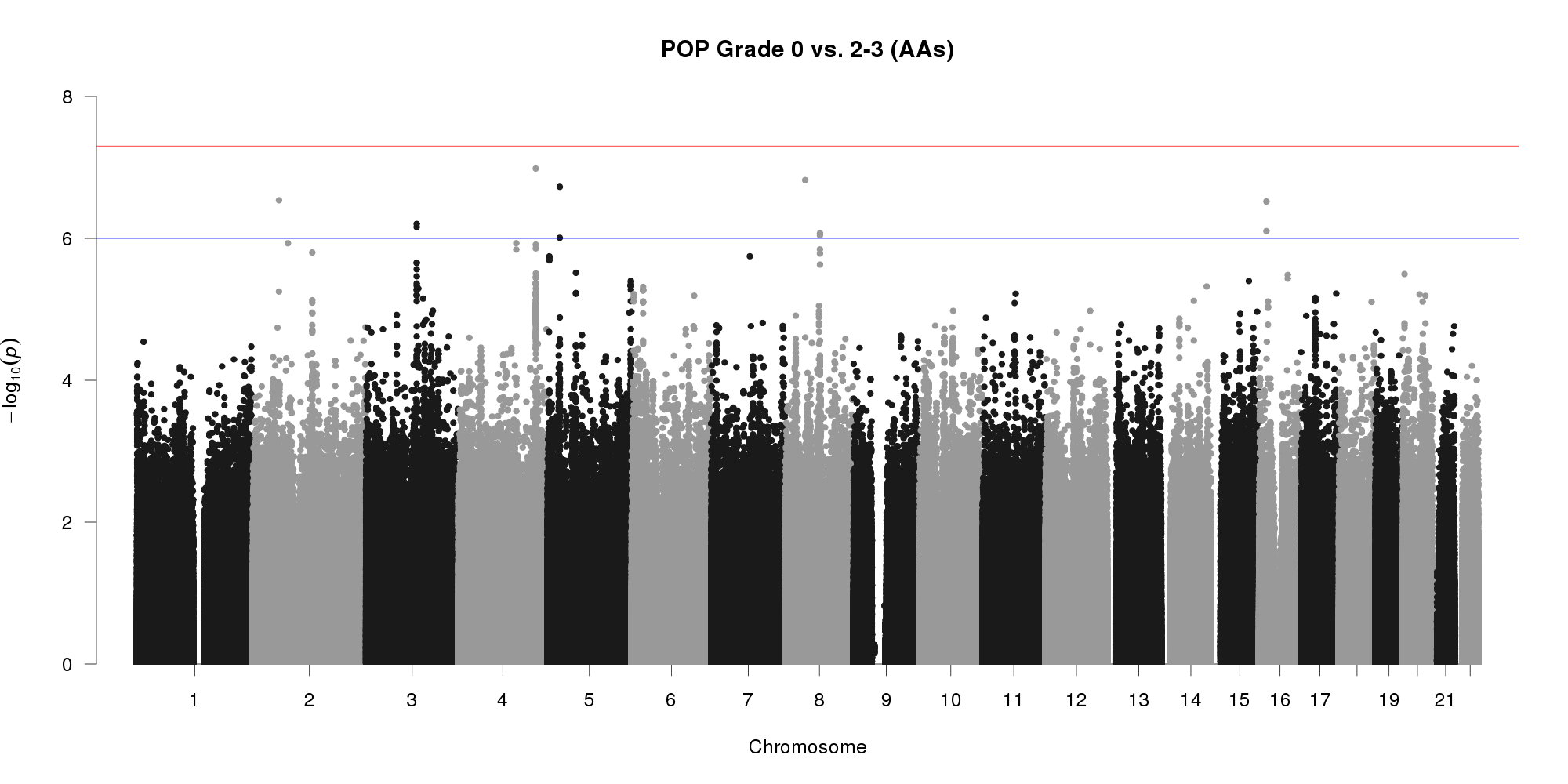

Supplement: S4 Fig — Logistic regression models were adjusted for age at ascertainment (continuous), body mass index (continuous), parity (continuous) and genetic ancestry components (continuous). X-axis: base-pair position; Y-axis: -log10(p-values). (TIF) [file pone.0141647.s004.tif]

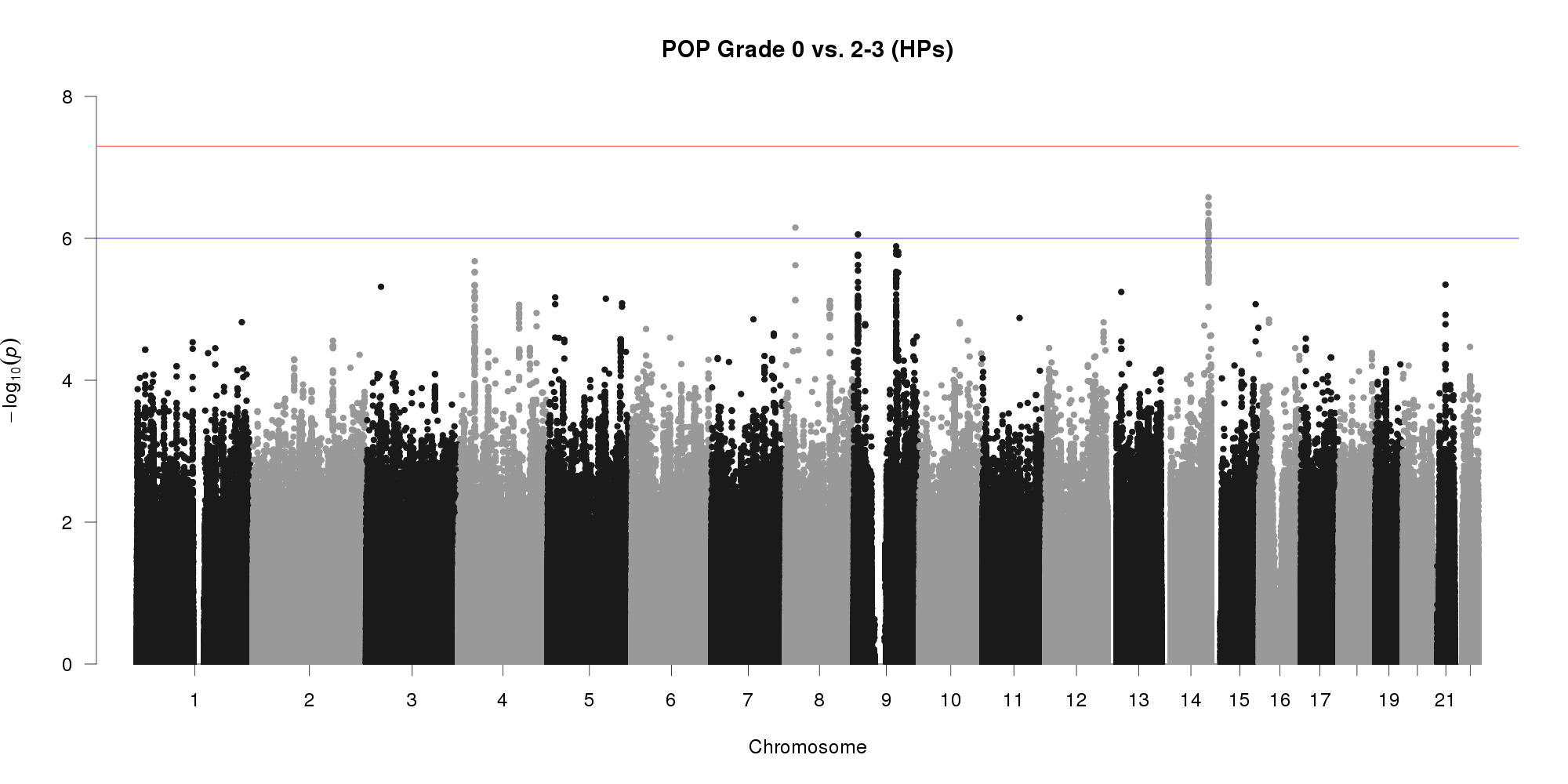

Supplement: S5 Fig — Logistic regression models were adjusted for age at ascertainment (continuous), body mass index (continuous), parity (continuous) and genetic ancestry components (continuous). X-axis: base-pair position; Y-axis: -log10(p-values). (TIF) [file pone.0141647.s005.tif]

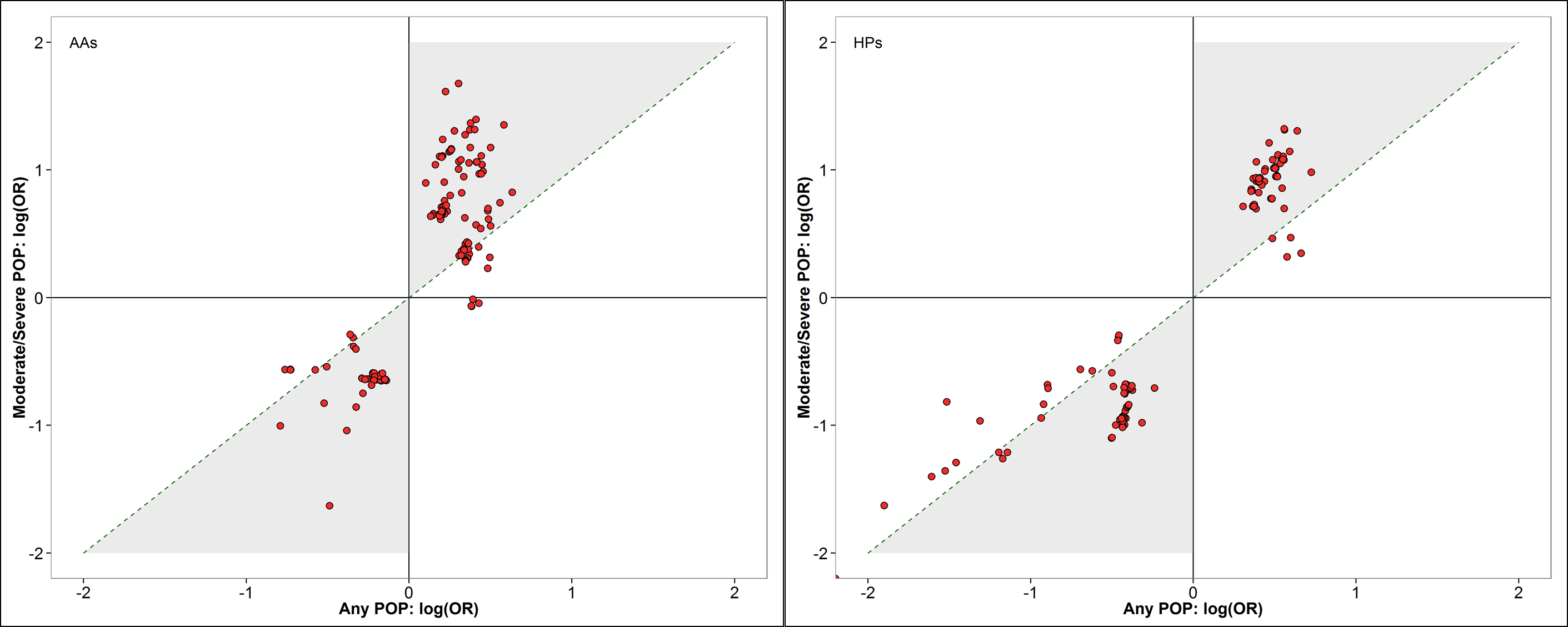

Supplement: S6 Fig — This figure compares beta-estimates of top hits (p <10–5) originating from either any POP or moderate/severe POP models where all models were adjusted for age, BMI, parity and ancestry components. X-axis and Y-axis represents natural log transformed odds ratios from any POP and moderate/severe POP models, respectively. left plot: AAs; right plot: HPs. (TIF) [file pone.0141647.s006.tif]

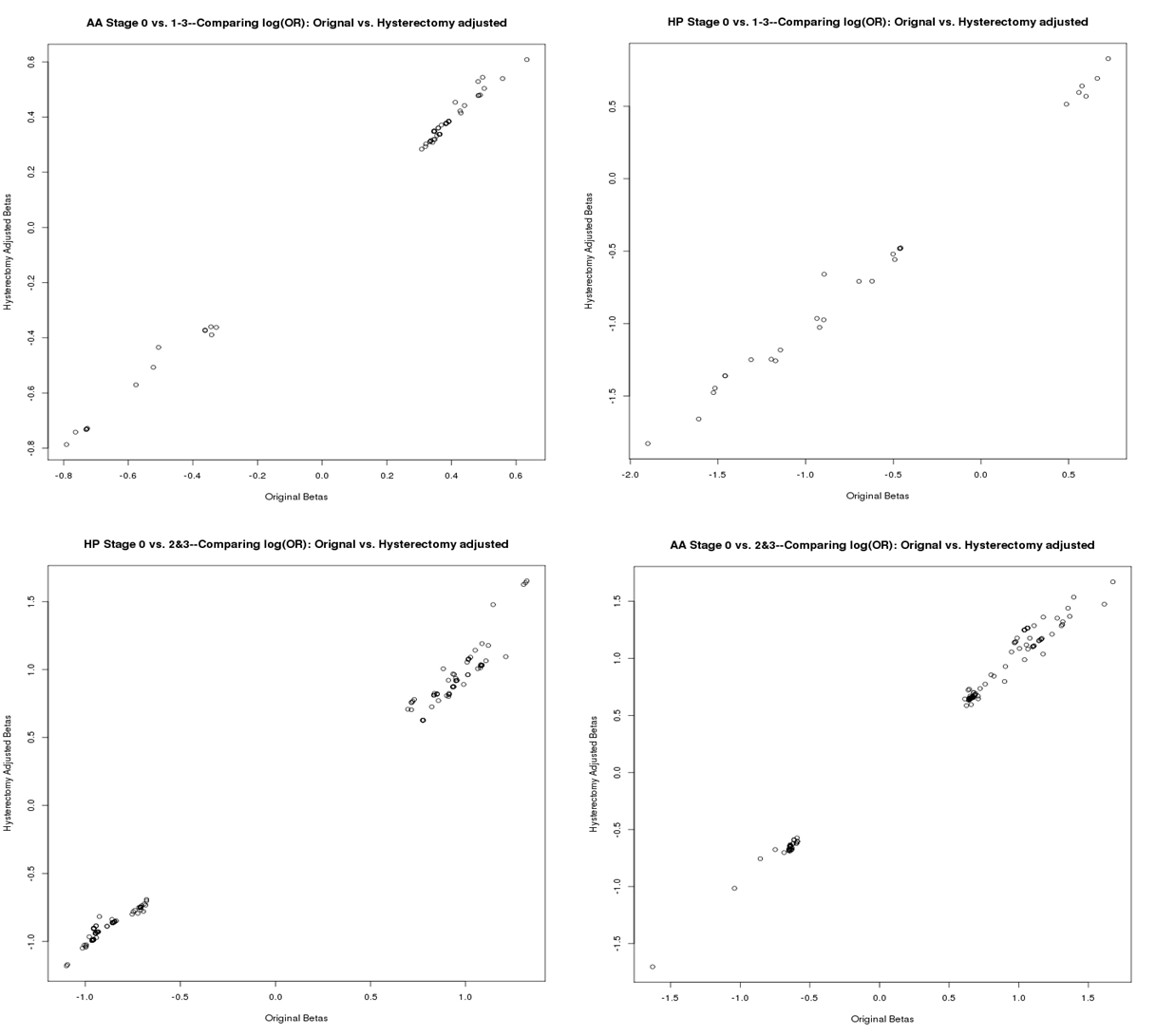

Supplement: S7 Fig — This figure compares beta-estimates of top hits (p < 10−5) from models adjusted for age, BMI, parity and ancestry components to models adjusted for hysterectomy in addition to the aforementioned variables. Original refers to betas from models adjusted for age, BMI, parity and ancestry components; Hysterectomy adjusted refers to betas from models adjusted for hysterectomy status in addition to factors in the original models. (TIF) [file pone.0141647.s007.tif]

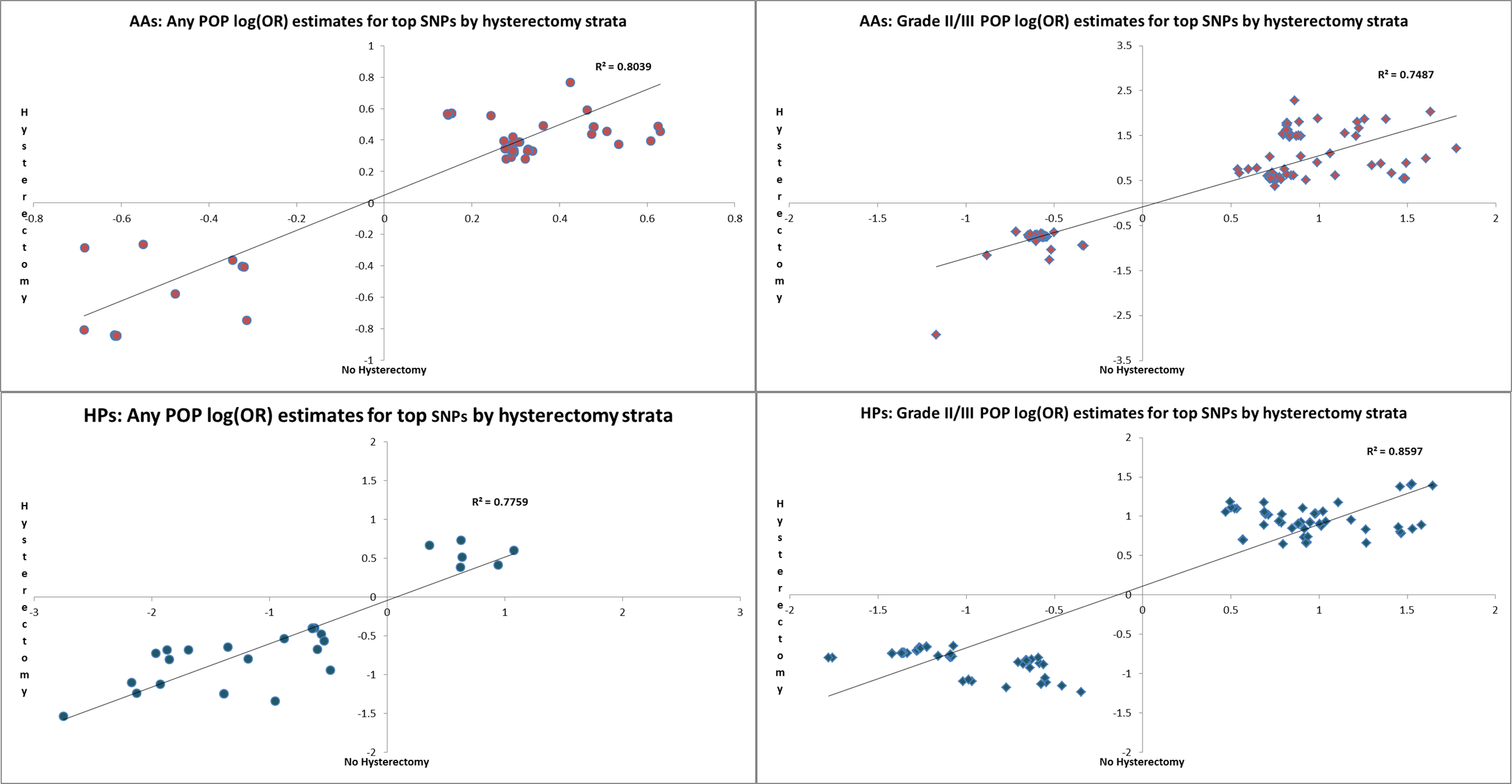

Supplement: S8 Fig — This figure compares beta-estimates of top hits (p < 10−5) from models adjusted for age, BMI, parity and ancestry components in women with and without a hysterectomy at baseline. X-axis and Y-axis represents natural log transformed odds ratios from analyses in women without hysterectomy and women with hysterectomy, respectively. Top 2 plots are for AAs (Any POP and moderate/severe POP, left to right). Bottom two plots are for HPs (Any POP and moderate/severe POP, left to right). (TIF) [file pone.0141647.s008.tif]
