# Supplementary material for: Genetic Determinants of Pelvic Organ Prolapse among African American and Hispanic Women in the Women’s Health Initiative
Source: PLoS One. 2015 Nov 6;10(11):e0141647. doi: 10.1371/journal.pone.0141647 (PMC4636147; doi:10.1371/journal.pone.0141647)
Supplement: S1 Table — The following table presents the associations between key risk factors in relation to any POP (grades 1–3) and severe/moderate POP (grades 2–3). (DOCX) [file pone.0141647.s009.docx]

**S1 Table. Associations between key risk factors for any pelvic organ prolapse and grades 2-3 pelvic organ prolapse in the WHI Hormone Therapy trial.**

| **Covariate** | **N**  **Any POP Cases/**  **Controls** | **Any Prolapse** | |  | **N  Grades 2-3 cases** | **Grades 2-3 Prolapse** | |
| --- | --- | --- | --- | --- | --- | --- | --- |
|  |  | **OR** | **95% CI** |  |  | **OR** | **95% CI** |
| Age (years) - continuous | 1399/1253 | 1.02 | (1.00-1.03) |  | 317 | 1.03 | (1.01-1.05) |
| Race/ethnicity |  |  |  |  |  |  |  |
| African American | 793/948 | 1.00 | (Referent) |  | 154 | 1.00 | (Referent) |
| Hispanic | 606/305 | 1.9 | (1.58-2.29) |  | 163 | 2.51 | (1.87-3.38) |
| BMI |  |  |  |  |  |  |  |
| <25 kg/m2 | 205/204 | 1.00 | (Referent) |  | 44 | 1.00 | (Referent) |
| 25 to 29.9 kg/m2 | 486/413 | 1.44 | (1.11-1.87) |  | 110 | 1.42 | (0.92-2.20) |
| ≥30 kg/m2 | 708/636 | 1.57 | (1.22-2.02) |  | 163 | 1.59 | (1.04-2.43) |
| Parity |  |  |  |  |  |  |  |
| Never pregnant | 120/193 | 1.00 | (Referent) |  | 12 | 1.00 | (Referent) |
| 1 | 134/180 | 1.1 | (0.78-1.56) |  | 19 | 1.67 | (0.76-3.66) |
| 2 | 294/272 | 1.64 | (1.21-2.22) |  | 56 | 3.17 | (1.60-6.26) |
| 3 | 251/236 | 1.46 | (1.07-1.99) |  | 50 | 2.92 | (1.46, 5.83) |
| 4 | 225/140 | 2.31 | (1.65-3.24) |  | 51 | 5.19 | (2.57-10.49) |
| ≥5 | 375/232 | 2.22 | (1.63-3.01) |  | 129 | 6.75 | (3.50-13.01) |
| Hysterectomy status |  |  |  |  |  |  |  |
| No | 896/382 | 1.00 | (Referent) |  | 228 | 1.00 | (Referent) |
| Yes | 503/871 | 0.24 | (0.20-0.29) |  | 89 | 0.17 | (0.12-0.22) |

BMI = body mass index; kg/m2 = kilograms per meters squared; OR = Odds Ratio; 95% CI = 95% Confidence Interval; The same set of individuals served as controls in both the Any POP and Grade 2-3 POP analyses.
