# Supplementary material for: Genetic Determinants of Pelvic Organ Prolapse among African American and Hispanic Women in the Women’s Health Initiative
Source: PLoS One. 2015 Nov 6;10(11):e0141647. doi: 10.1371/journal.pone.0141647 (PMC4636147; doi:10.1371/journal.pone.0141647)
Supplement: S2 Table — The following table presents results on single nucleotide polymorphisms (SNPs) associated with Grade 1–3 prolapse, which had p < 0.00001 in the fixed effects meta-analysis. Results from random-effects models and Heterogeneity Scores (I2) are also presented. (DOCX) [file pone.0141647.s010.docx]

**S2 Table. Results from meta-analysis of Grade 0 vs. Grade 1-3 POP across African American (AA) and Hispanic (HP) Women from the Women’s Health Initiative (p < 0.00001).**

| **SNP** | **CHR** | **BP** | **On/Nearby genes** | **EA/RA** | **African American** | | | | | **Hispanic** | | | | | **Meta-analysis** | | |
| --- | --- | --- | --- | --- | --- | --- | --- | --- | --- | --- | --- | --- | --- | --- | --- | --- | --- |
|  |  |  |  |  | **EAF** | **Info** | **OR** | **P** | **N** | **EAF** | **Info** | **OR** | **P** | **N** | **OR** | **P** | **I^2^** |
| rs1335802 | 6 | 134518512 | *SGK1** | C/G | 0.58 | 0.91 | 1.41 | 6.62x10^-6^ | 1741 | 0.39 | 0.99 | 1.22 | 5.71x10^-2^ | 911 | 1.34 | 1.99x10^-6^ | 24 |
| rs12122043 | 1 | 208789949 | *RPS26P13* | G/A | 0.09 | 0.99 | 1.59 | 4.25x10^-5^ | 1741 | 0.16 | 0.99 | 1.39 | 1.15x10^-2^ | 911 | 1.50 | 2.07x10^-6^ | 0 |
| rs12018439 | 13 | 85391073 | *LINC00333* | T/G | 0.37 | 1.00 | 0.74 | 4.38x10^-5^ | 1741 | 0.36 | 1.00 | 0.77 | 1.47x10^-2^ | 911 | 0.75 | 2.07x10^-6^ | 0 |
| rs10793273 | 11 | 77742595 | *NDUFC2-KCTD14** | C/T | 0.19 | 0.95 | 0.72 | 5.44x10^-4^ | 1741 | 0.37 | 0.98 | 0.71 | 1.16x10^-3^ | 911 | 0.71 | 2.11x10^-6^ | 0 |
| rs10776501 | 4 | 124387479 | *SPRY1, SPATA5* | A/G | 0.69 | 1.00 | 1.35 | 8.26x10^-5^ | 1741 | 0.83 | 1.00 | 1.51 | 6.86x10^-3^ | 911 | 1.38 | 2.20x10^-6^ | 0 |
| rs61173913 | 2 | 201351874 | *KCTD18, SPATS2L* | T/TA | 0.13 | 0.97 | 0.67 | 4.24x10^-4^ | 1741 | 0.22 | 0.98 | 0.66 | 1.78x10^-3^ | 911 | 0.67 | 2.50x10^-6^ | 0 |
| rs6669972 | 1 | 30330967 | *RP11-111I12.1* | T/C | 0.09 | 0.93 | 0.70 | 1.13x10^-2^ | 1741 | 0.24 | 0.95 | 0.58 | 5.67x10^-5^ | 911 | 0.64 | 3.24x10^-6^ | 0 |
| rs56156301 | 8 | 104147804 | *BAALCOS* LINC01181* | A/G | 0.10 | 0.96 | 0.64 | 7.06x10^-4^ | 1741 | 0.11 | 0.99 | 0.56 | 1.49x10^-3^ | 911 | 0.61 | 4.17x10^-6^ | 0 |
| rs7147087 | 14 | 59236307 | *RPL31P4, XR_429367.1* | G/T | 0.08 | 1.00 | 0.72 | 1.52x10^-2^ | 1741 | 0.17 | 1.00 | 0.53 | 3.14x10^-5^ | 911 | 0.63 | 4.57x10^-6^ | 55 |
| rs13289122 | 9 | 107757003 | *XR_242662.1, ABCA1* | T/C | 0.47 | 0.99 | 1.41 | 9.06x10^-7^ | 1741 | 0.48 | 0.99 | 1.10 | 3.49x10^-1^ | 911 | 1.30 | 5.27x10^-6^ | 77 |
| rs10826468 | 10 | 28709605 | *RPSAP10* | C/T | 0.41 | 0.98 | 0.77 | 2.02x10^-4^ | 1741 | 0.51 | 1.00 | 0.76 | 8.49x10^-3^ | 911 | 0.77 | 5.28x10^-6^ | 0 |
| rs72636617 | 13 | 95075219 | *DCT, GPC6* | A/G | 0.09 | 0.97 | 1.51 | 2.03x10^-4^ | 1741 | 0.12 | 0.98 | 1.45 | 8.87x10^-3^ | 911 | 1.49 | 5.64x10^-6^ | 0 |
| rs2236521 | 20 | 60892116 | *LAMA5** | A/G | 0.16 | 0.93 | 1.49 | 1.84x10^-5^ | 1741 | 0.49 | 0.95 | 1.23 | 4.59x10^-2^ | 911 | 1.37 | 6.03x10^-6^ | 46 |
| rs181766936 | 3 | 158729443 | *IQCJ-SCHIP1* | T/C | 0.20 | 0.94 | 0.69 | 7.71x10^-5^ | 1741 | 0.07 | 0.95 | 0.60 | 2.41x10^-2^ | 911 | 0.68 | 6.40x10^-6^ | 0 |
| rs72655104 | 13 | 98823146 | *FARP1** | G/A | 0.14 | 0.90 | 1.45 | 1.51x10^-4^ | 1741 | 0.10 | 0.98 | 1.45 | 1.49x10^-2^ | 911 | 1.45 | 6.66x10^-6^ | 0 |
| rs2019216 | 17 | 21909650 | *FLJ36000* | T/C | 0.64 | 1.00 | 0.83 | 8.02x10^-3^ | 1741 | 0.59 | 1.00 | 0.67 | 5.06x10^-5^ | 911 | 0.77 | 6.81x10^-6^ | 69 |
| rs6947471 | 7 | 154381475 | *DPP6* | T/A | 0.43 | 0.98 | 1.29 | 2.61x10^-4^ | 1741 | 0.15 | 0.97 | 1.45 | 6.38x10^-3^ | 911 | 1.32 | 7.01x10^-6^ | 0 |
| rs7290192 | 22 | 49318526 | *LOC100128946* | T/A | 0.07 | 0.85 | 0.50 | 4.37x10^-5^ | 1741 | 0.12 | 0.81 | 0.67 | 2.79x10^-2^ | 911 | 0.57 | 7.08x10^-6^ | 27 |
| rs200147305 | 13 | 49431295 | *PSME2P2, FNDC3A* | T/TAC | 0.67 | 0.94 | 1.30 | 6.57x10^-4^ | 1741 | 0.74 | 0.90 | 1.46 | 3.05x10^-3^ | 911 | 1.34 | 8.56x10^-6^ | 0 |
| rs12348212 | 9 | 1221968 | *RPS27AP14* | A/G | 0.11 | 0.94 | 1.55 | 3.64x10^-5^ | 1741 | 0.06 | 0.98 | 1.39 | 9.56x10^-2^ | 911 | 1.52 | 9.71x10^-6^ | 0 |
| rs7172133 | 15 | 96025311 | *LINC00924** | C/A | 0.13 | 1.00 | 1.50 | 3.35x10^-5^ | 1741 | 0.27 | 1.00 | 1.24 | 4.52x10^-2^ | 911 | 1.38 | 9.87x10^-6^ | 41 |

SNP=single nucleotide polymorphism; CHR=chromosome; BP=base pair; EA=effect allele; RA=reference allele; EAF=effect allele frequency; Info=Imputation quality criteria; OR=odds ratio; P=p-value; N=sample size; *SNP is on gene
