# Supplementary material for: Genetic Determinants of Pelvic Organ Prolapse among African American and Hispanic Women in the Women’s Health Initiative
Source: PLoS One. 2015 Nov 6;10(11):e0141647. doi: 10.1371/journal.pone.0141647 (PMC4636147; doi:10.1371/journal.pone.0141647)
Supplement: S3 Table — The following table presents results on single nucleotide polymorphisms (SNPs) associated with Grade 2–3 prolapse, which had p < 0.00001 in the fixed effects meta-analysis. Results from random-effects models and Heterogeneity Scores (I2) are also presented. (DOCX) [file pone.0141647.s011.docx]

**S3 Table. Results from meta-analysis of Grade 0 vs. Grade 2-3 POP across African American (AA) and Hispanic (HP) women from the Women’s Health Initiative (p < 0.00001).**

| **SNP** | **CHR** | **BP** | **On/Nearby genes** | **EA/RA** | **African American** | | | | | **Hispanic** | | | | | **Meta-analysis** | | |
| --- | --- | --- | --- | --- | --- | --- | --- | --- | --- | --- | --- | --- | --- | --- | --- | --- | --- |
|  |  |  |  |  | **EAF** | **Info** | **OR** | **P** | **N** | **EAF** | **Info** | **OR** | **P** | **N** | **OR** | **P** | **I^2^** |
| rs1305024 | 9 | 91809082 | *SHC3* | C/T | 0.31 | 1.00 | 0.65 | 1.88x10^-3^ | 1102 | 0.49 | 1.00 | 0.54 | 1.10x10^-4^ | 468 | 0.60 | 1.03x10^-6^ | 0 |
| rs1378770 | 7 | 114363566 | *FOXP2* | G/A | 0.15 | 1.00 | 1.72 | 2.03x10^-3^ | 1102 | 0.21 | 1.00 | 1.89 | 2.54x10^-4^ | 468 | 1.80 | 1.83x10^-6^ | 0 |
| rs7308116 | 12 | 108200596 | *LOC101929127, ASCL4* | A/G | 0.64 | 1.00 | 1.53 | 1.72x10^-3^ | 1102 | 0.41 | 1.00 | 1.74 | 3.00x10^-4^ | 468 | 1.61 | 2.10x10^-6^ | 0 |
| rs17459044 | 9 | 131853624 | *DOLPP1, CRAT* | T/C | 0.38 | 0.94 | 0.68 | 4.09x10^-3^ | 1102 | 0.63 | 0.98 | 0.55 | 1.01x10^-4^ | 468 | 0.62 | 2.23x10^-6^ | 0 |
| rs28573326 | 4 | 166338328 | *CPE** | G/A | 0.29 | 0.97 | 2.14 | 1.04x10^-7^ | 1102 | 0.26 | 0.99 | 1.18 | 3.28x10^-1^ | 468 | 1.67 | 2.49x10^-6^ | 86 |
| rs112881630 | 6 | 6722957 | *LOC101928047** | G/A | 0.14 | 0.80 | 2.04 | 4.29x10^-4^ | 1102 | 0.06 | 0.87 | 2.59 | 1.76x10^-3^ | 468 | 2.19 | 3.11x10^-6^ | 0 |
| rs1335802 | 6 | 134518512 | *SGK1** | C/G | 0.58 | 0.91 | 1.87 | 6.44x10^-6^ | 1102 | 0.39 | 0.98 | 1.34 | 5.40x10^-2^ | 468 | 1.61 | 3.62x10^-6^ | 62 |
| rs3850352 | 2 | 56255496 | *MIR216B* | C/T | 0.82 | 0.95 | 0.42 | 2.91x10^-7^ | 1102 | 0.53 | 0.95 | 0.79 | 1.03x10^-1^ | 468 | 0.60 | 4.14x10^-6^ | 87 |
| rs12692119 | 2 | 127621089 | *GYPC, BIN1* | C/A | 0.56 | 1.00 | 0.53 | 1.59x10^-6^ | 1102 | 0.88 | 1.00 | 0.83 | 3.90x10^-1^ | 468 | 0.59 | 5.02x10^-6^ | 66 |
| rs11231420 | 11 | 55644734 | *TRIM51, LOC100507561* | C/T | 0.32 | 0.96 | 0.59 | 1.60x10^-4^ | 1102 | 0.25 | 1.00 | 0.64 | 9.65x10^-3^ | 468 | 0.61 | 5.12x10^-6^ | 0 |
| rs1584120 | 12 | 23599253 | *SOX5* | T/C | 0.62 | 0.97 | 1.63 | 4.48x10^-4^ | 1102 | 0.73 | 0.99 | 1.65 | 4.46x10^-3^ | 468 | 1.64 | 6.27x10^-6^ | 0 |
| rs34333482 | 6 | 116855952 | *FAM26D** | AG/A | 0.48 | 0.99 | 0.60 | 6.07x10^-5^ | 1102 | 0.59 | 0.99 | 0.72 | 2.30x10^-2^ | 468 | 0.65 | 6.28x10^-6^ | 0 |
| rs311392 | 8 | 55084782 | *CHCHD2P10, LYPLA1* | C/T | 0.75 | 1.00 | 1.78 | 9.39x10^-5^ | 1102 | 0.55 | 1.00 | 1.45 | 1.42x10^-2^ | 468 | 1.61 | 6.58x10^-6^ | 0 |
| rs7623488 | 3 | 141434403 | *RNF7, TPT1P3* | C/T | 0.50 | 0.98 | 0.57 | 1.15x10^-5^ | 1102 | 0.26 | 0.99 | 0.76 | 1.09x10^-1^ | 468 | 0.63 | 7.34x10^-6^ | 41 |
| rs11525841 | 7 | 85183168 | *LOC729630* | A/G | 0.12 | 1.00 | 2.33 | 1.73x10^-5^ | 1102 | 0.18 | 1.00 | 1.46 | 3.46x10^-2^ | 468 | 1.81 | 8.34x10^-6^ | 67 |
| rs6678723 | 1 | 232156245 | *DISC1*, TSNAX-DISC1* | A/G | 0.22 | 1.00 | 0.55 | 8.78x10^-5^ | 1102 | 0.16 | 1.00 | 0.64 | 2.83x10^-2^ | 468 | 0.58 | 8.40x10^-6^ | 0 |
| rs7007076 | 8 | 124730029 | *ANXA13** | C/G | 0.21 | 0.89 | 1.63 | 2.53x10^-3^ | 1102 | 0.08 | 0.94 | 2.41 | 4.29x10^-4^ | 468 | 1.84 | 8.53x10^-6^ | 41 |
| rs13418288 | 2 | 177016012 | *HOXD3*, HOXD4* | T/C | 0.38 | 0.96 | 0.57 | 5.33x10^-5^ | 1102 | 0.10 | 0.98 | 0.62 | 6.02x10^-2^ | 468 | 0.58 | 8.65x10^-6^ | 0 |
| rs202229006 | 1 | 18801855 | *KLHDC7A* | T/TG | 0.12 | 0.87 | 0.46 | 3.15x10^-4^ | 1102 | 0.08 | 0.77 | 0.43 | 8.95x10^-3^ | 468 | 0.45 | 8.70x10^-6^ | 0 |
| rs512279 | 18 | 58232100 | *LOC100421385* | C/T | 0.59 | 0.89 | 1.69 | 1.19x10^-4^ | 1102 | 0.68 | 0.85 | 1.47 | 2.25x10^-2^ | 468 | 1.60 | 9.48x10^-6^ | 0 |
| rs11743712 | 5 | 64851665 | *CENPK** | T/A | 0.22 | 1.00 | 1.84 | 6.32x10^-5^ | 1102 | 0.23 | 1.00 | 1.45 | 3.12x10^-2^ | 468 | 1.66 | 9.68x10^-6^ | 7 |

SNP=single nucleotide polymorphism; CHR=chromosome; BP=base pair; EA=effect allele; RA=reference allele; EAF=effect allele frequency; Info=Imputation quality criteria; OR=odds ratio; P=p-value; N=sample size; *SNP is on gene
